# Cell-type-specific aging clocks to quantify aging and rejuvenation in neurogenic regions of the brain

**DOI:** 10.1038/s43587-022-00335-4

**Published:** 2022-12-19

**Authors:** Matthew T. Buckley, Eric D. Sun, Benson M. George, Ling Liu, Nicholas Schaum, Lucy Xu, Jaime M. Reyes, Margaret A. Goodell, Irving L. Weissman, Tony Wyss-Coray, Thomas A. Rando, Anne Brunet

**Affiliations:** 1 Department of Genetics, Stanford University, Stanford, CA USA; 2 Genetics Graduate Program, Stanford University, Stanford, CA USA; 3 Biomedical Informatics Graduate Program, Stanford University, Stanford, CA USA; 4 Stanford Medical Scientist Training Program, Stanford University, Stanford, CA USA; 5 Institute for Stem Cell Biology and Regenerative Medicine, Stanford University, Stanford, CA USA; 6 Department of Neurology and Neurological Sciences, Stanford University School of Medicine, Stanford, CA USA; 7 Department of Biology, Stanford University, Stanford, CA USA; 8 Stem Cells and Regenerative Medicine Center, Baylor College of Medicine, Houston, TX USA; 9 Department of Molecular and Human Genetics, Baylor College of Medicine, Houston, TX USA; 10 Department of Molecular and Cellular Biology, Baylor College of Medicine, Houston, TX USA; 11 Ludwig Center for Cancer Stem Cell Research and Medicine, Stanford University School of Medicine, Stanford, CA USA; 12 Stanford Cancer Institute, Stanford University School of Medicine, Stanford, CA USA; 13 Department of Pathology, Stanford University School of Medicine, Stanford, CA USA; 14 Wu Tsai Neurosciences Institute, Stanford University, Stanford, CA USA; 15 Glenn Center for the Biology of Aging, Stanford University, Stanford, CA USA; 16 Neurology Service, Veterans Affairs Palo Alto Health Care System, Palo Alto, CA USA; 17 Present Address: Department of Neurology, UCLA, Los Angeles, CA USA; 18 Present Address: Broad Stem Cell Research Center, UCLA, Los Angeles, CA USA

**Keywords:** Gene expression, Machine learning

## Abstract

The diversity of cell types is a challenge for quantifying aging and its reversal. Here we develop ‘aging clocks’ based on single-cell transcriptomics to characterize cell-type-specific aging and rejuvenation. We generated single-cell transcriptomes from the subventricular zone neurogenic region of 28 mice, tiling ages from young to old. We trained single-cell-based regression models to predict chronological age and biological age (neural stem cell proliferation capacity). These aging clocks are generalizable to independent cohorts of mice, other regions of the brains, and other species. To determine if these aging clocks could quantify transcriptomic rejuvenation, we generated single-cell transcriptomic datasets of neurogenic regions for two interventions—heterochronic parabiosis and exercise. Aging clocks revealed that heterochronic parabiosis and exercise reverse transcriptomic aging in neurogenic regions, but in different ways. This study represents the first development of high-resolution aging clocks from single-cell transcriptomic data and demonstrates their application to quantify transcriptomic rejuvenation.

## Abstract (web summary)

Single-cell transcriptomic data from a neurogenic region of the mouse brain were used to build aging clocks for specific neural cell types. These clocks showed that heterochronic parabiosis and exercise lead to distinct transcriptomic rejuvenation patterns across cell types.

## Main

Aging is the progressive deterioration of cellular and organismal function. Age-dependent decline is linked in large part to the passage of time and therefore the chronological age of an individual. But such decline is not inexorable. At the same chronological age, some individuals have better organismal and tissue fitness (biological age) than others. Furthermore, aging trajectories can be slowed, and some aspects of aging can be reversed by specific interventions, including dietary restriction, exercise, reprogramming factors, senolytic compounds and young blood factors^1–6^. As aging is the primary risk factor for many diseases, particularly neurodegenerative diseases^7,8^, a better understanding of aging and ‘rejuvenation’ strategies could yield large benefits for a wide range of diseases.

Aging is complex and difficult to quantify. One quantification approach is to use machine learning to build age prediction models—‘aging clocks’—which can serve as integrative aging biomarkers. Such clocks should also accelerate our understanding of existing interventions and help identify new strategies to counter aging and age-related diseases. Machine learning models trained on high-dimensional datasets (for example, DNA methylation, transcriptomics and proteomics) can predict chronological age with remarkable accuracy. For example, regression-based aging clocks trained on DNA methylation profiles from multiple tissues (‘epigenetic aging clocks’)^9–13^ or blood plasma protein profiles^14–17^ have striking performance to predict chronological age in humans. Aging clocks directly optimized to predict biological age have also been developed on functional phenotypes^12,13,18^ or time remaining until death^19,20^. Interestingly, beneficial health interventions such as diet and exercise^21–23^ and genetic manipulations^24–26^ result in younger predictions from epigenetic aging clocks trained on chronological age. Thus, epigenetic aging clocks, despite being trained on chronological age, also capture dimensions of biological age.

So far, molecular aging clocks have largely relied on datasets built using bulk tissue input or purified cell populations^9–13,27–34^. Bulk tissue profiles (and even purified populations) average the molecular profiles from many cells, integrating tissue composition changes and cell-type-specific responses. Hence, the cell-type-specific contributions to aging and rejuvenation detected by these clocks remain unclear. While single-cell DNA methylation and transcriptomic data have started to be used to classify age^35–37^, cell-type-specific transcriptomic aging clocks have not yet been generated. Thus, it remains to be determined if aging clocks of different cell types ‘tick’ at different rates, which cell types predict age most accurately and how specific cell types respond to different interventions. The rapid advance of single-cell RNA-sequencing (RNA-seq) technologies provides a great opportunity to explore these unaddressed questions and identify new molecular aging clocks to study interventions to counter aging and age-related diseases.

## Results

### Cell-type-specific transcriptomic aging clocks

**Fig. 1:**
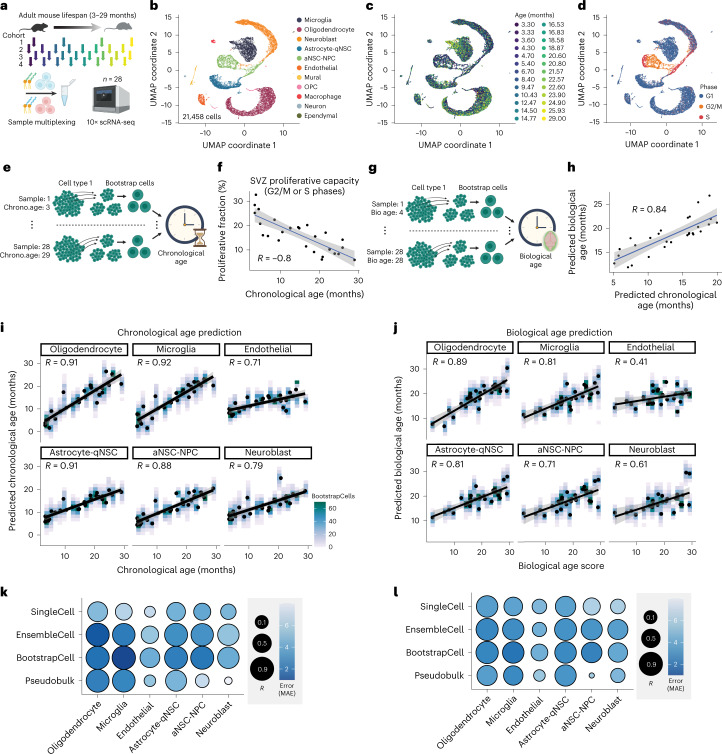
Cell-type-specific transcriptomic aging clocks for neurogenic regions. **a**, Training data for single-cell transcriptomic aging clocks. 10x Genomics single-cell transcriptomics on SVZ neurogenic regions from four independent cohorts of 4–8 male mice, aged 3.3 to 29 months (Supplementary Table 1). SVZ regions from the same cohort were multiplexed using LMO labeling (MULTI-seq). **b**, UMAP projection of 21,458 high-quality cells from SVZ single-cell transcriptomes across cohorts. Each dot represents the transcriptome of an individual cell with transcripts detected from at least 500 genes. **c**, Same as in **b** but colored by mouse age. Two pairs of mice had the same age, resulting in 26 age colors (28 mice). **d**, Same as in **b** but colored by the predicted cell cycle state based on Seurat’s CellCycleScoring function. **e**, Schematic depicting the generation of BootstrapCells for training chronological clocks. From each cell type and sample, 15 cells were sampled and combined to generate one BootstrapCell. This process was repeated 100 times per cell type and sample combination, to generate a training dataset that equally weighted each SVZ sample. **f**, SVZ proliferative fraction (cells predicted to be G2/M or S phase) as a function of chronological age. *R* represents Pearson’s correlation coefficient. The gray band corresponds to the 95% confidence interval. **g**, Schematic depicting the process of generating BootstrapCells for training biological age clocks. Biological age was defined as the SVZ proliferative fraction (**f**). **h**, Predicted biological age as a function of predicted chronological age. R represents Pearson’s correlation coefficient. Gray band corresponds to 95% confidence interval. **i**, Performance of BootstrapCell chronological age prediction across cell types. Density of BootstrapCell predictions is depicted in color and overlaid black dots represent the median prediction for each sample. Performance is based on cross-cohort validation. R values are Pearson’s correlation coefficients at the sample level. **j**, As in **i** but for BootstrapCell biological age score prediction across cell types. Biological age score is a linear transformation of the SVZ proliferative fraction. **k**, Overview of Pearson’s correlation coefficients and median absolute error (MAE) values for various methods of predicting chronological age across cell types. SingleCell uses bona fide single-cell transcriptomes with minimal processing as input to a lasso regression model. BootstrapCell uses the preprocessing method depicted in **e** and a lasso model. EnsembleCell involves repeatedly partitioning cells into groups of 15 cells and training an ensemble of elastic net models. Pseudobulk involves naïve pseudobulking all cells from the same cell type and sample and using a lasso regression model. Performance is based on cross-cohort validation. **l**, As in **k** but evaluating biological age prediction.

**Extended Data Fig. 1:**
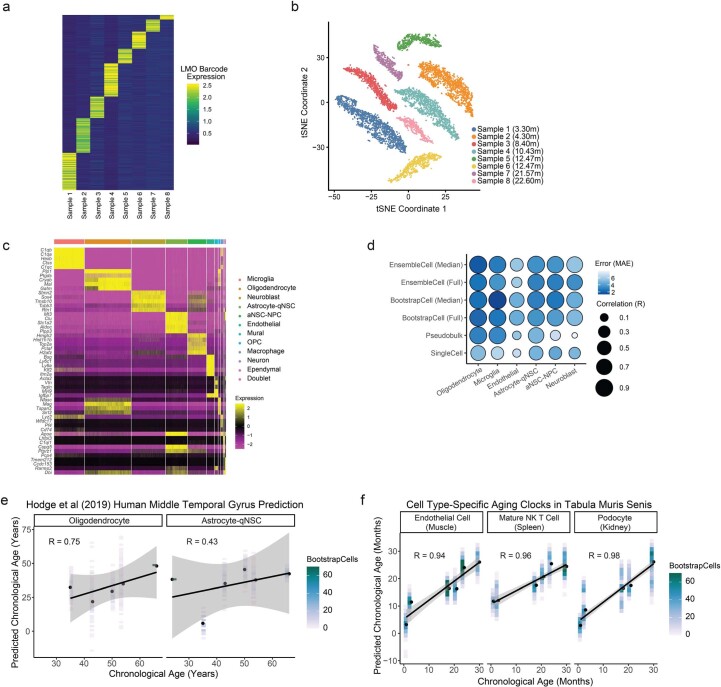
Characteristics of the SVZ single-cell transcriptomic data and generalization performance of cell-type-specific aging clocks to humans and generalization of the framework to other cell types and tissues. **a**, Lipid-modified oligonucleotide cell barcodes detected from 8 SVZ samples multiplexed in one 10x lane. **b**, Same as in (a) but visualized using tSNE. Samples 5 and 6 were from mice of the same age (and colored in the same color in Fig. 1c). **c**, Heatmap of single cell gene expression for top 5 cell type markers used for annotation of cell type clusters. Colored bar on top indicates the various cell type clusters. **d**, Overview of Pearson correlation coefficients (R) and median absolute error (MAE) values for tested methods of predicting chronological age across cell types from single-cell transcriptomic data, including both full distribution (Full) and median metrics only (Median). Performance is based on cross-cohort-validation. **e**, Correlation plot to assess the generalizability of chronological aging clocks (BoostrapCell) to a human dataset from Hodge et al^61^. the single-nucleus RNA-seq dataset of the middle temporal gyrus of human patients of different ages (Hodge et al.^61^). Density of BootstrapCell predictions is depicted in color and overlaid black dots represent the median prediction for each sample. R values are Pearson’s correlation coefficients at the sample level. Bands correspond to 95% confidence interval. **f**, Performance of chronological aging clocks (BootstrapCell) derived from the single cell RNA-seq multi-tissue atlas Tabula Muris Senis, 2020. Predicted chronological age of endothelial cells from limb muscle, mature natural killer (NK) T cells from spleen, and podocyte cells from kidney from aging clocks built on Tabula Muris Senis as a function of actual chronological age for several mice of different ages. Density of BootstrapCell predictions is depicted in color and overlaid black dots represent the median prediction for each sample. Performance is based on cross-mouse validation. R values are Pearson’s correlation coefficients at the sample level.

As a paradigm for tissue aging and functional decline in the brain, we focused on the neurogenic region located in the subventricular zone (SVZ) of the adult mammalian brain. The SVZ neurogenic region (or ‘niche’) contains neural stem cells (NSCs) that give rise to differentiated cells (neurons, astrocytes) that are important for olfactory discrimination and repair upon injury^38–45^. Importantly, this neurogenic region contains at least 11 different cell types and experiences age-related changes correlated with deterioration in tissue function^42,46–49^. We built cell-type-specific aging clocks trained to predict the chronological or biological age of the SVZ neurogenic niche. To train these clocks, we performed single-cell RNA-seq on neurogenic regions from 28 mice, tiling 26 different ages from 3 months (young adult) to 29 months (geriatric adult; Fig. 1a). Given the constraining cost of single-cell RNA-seq, we used lipid-modified oligonucleotide (LMO) labeling (MULTI-seq; Methods)^50^ to multiplex samples within four independent cohorts, each with 4–8 mice (Supplementary Table 1). After demultiplexing and quality control, we obtained 21,458 high-quality single-cell transcriptomes (Extended Data Fig. 1a,b). Clustering and uniform manifold approximation and projection (UMAP) visualization confirmed the presence of 11 cell types in this neurogenic region, including differentiated cell types (microglia, endothelial cells, oligodendrocytes) and cells from the NSC lineage (astrocytes and quiescent neural stem cells (qNSCs), activated neural stem cells (aNSCs), neural progenitor cells (NPCs) and neuroblasts; Fig. 1b, Extended Data Fig. 1c and Supplementary Table 2). This analysis also corroborated the decline of proliferating NSCs in this region during aging (Fig. 1c,d,f)^42^. Our dataset provides a high temporal resolution resource to characterize aging in a neurogenic region in the brain.

To develop robust single-cell-based aging clocks, we focused on the six most abundant recovered cell types in the SVZ neurogenic region—oligodendrocytes, microglia, endothelial cells, astrocytes-qNSCs (which cluster together; Fig. 1b and Methods), aNSC-NPCs (which cluster together; Fig. 1b and Methods) and neuroblasts. We first developed chronological age models that maximize correlation and minimize error between predicted and true chronological age. We built different models (lasso and elastic net regression^51–53^) from single-cell transcriptomic data for each of the six cell types as an input (Methods). We evaluated the performance of the models on true chronological age, by building models on 3 of the 4 cohorts and validation was performed on the remaining cohort (cross-cohort validation). This strategy avoids performance inflation caused by training and evaluating on correlated cells from the same animal or animals from the same cohort. Our resulting top-performing chronological aging clocks, termed ‘bootstrap’ and ‘ensemble’, are groups of lasso and elastic net models trained on either bootstrap-sampled or randomly partitioned and merged meta cells, termed BootstrapCells or EnsembleCells (Methods). For the bootstrap models, 100 BootstrapCells were generated by taking 100 random samples of 15 cells for each cell type and animal combination, such that each animal contributed equally to the training data (Fig. 1e). For the ensemble model, a random partitioning and elastic net model training process was repeated 20 times and then combined to generate a single ensemble model for a given cell type, such that each cell contributed equally. In our cross-cohort validation, these two models performed well to predict chronological age. For example, oligodendrocyte bootstrap models predicted chronological age with a correlation *R* = 0.91 and an error = 1.6 months and microglia Bootstrap models predicted chronological age with a correlation *R* = 0.92 and an error = 2.1 months (Fig. 1i and Supplementary Table 3). Such performance in a cross-cohort validation scheme suggests that these chronological aging clocks are not batch dependent. Overall, these models had *R* values ranging from 0.71 to 0.92 and errors ranging from 1.6 to 5.4 months, and they uniformly surpassed the performance of raw single-cell trained clocks and pseudobulked clocks (that is, pool of all cells from each cell type; Fig. 1k, Extended Data Fig. 1d, Supplementary Table 3 and Methods).

We also developed ‘biological aging clocks’ from our single-cell transcriptomic data–that is, clocks that are trained on a functional metric of the tissue, rather than chronological age. While chronological aging clocks can record aspects of biological age^15,22,24^ and predict disease probability^54,55^, clocks trained on functional metrics linked to biological age may be particularly useful for intervention assessment^12,24^. The primary functional role of the SVZ neurogenic region is to harbor proliferating NSCs that can produce new neurons, which in turn integrate into functional neural circuits^39,42–45^. The proliferative capacity of NSCs in the SVZ neurogenic region declines with age, and this decline may be considered a functional metric of biological aging of this region^56–59^. To define neurogenic region ‘fitness’, we quantified the proliferative fraction of cells (consisting almost exclusively of aNSC-NPCs and neuroblasts) in the neurogenic regions from each of the 28 mice, based on cell cycle signatures (Fig. 1d). The fraction of cells predicted to be proliferative (in G2/M and S phases, the ‘proliferative fraction’) decreased with age, as expected (Fig. 1f). Here we used proliferative fraction as a functional metric of the neurogenic region, which we defined as ‘biological age’. We trained a suite of clocks analogous to those described above, except using aNSC-NPC proliferative fraction as biological age (Fig. 1g). These biological aging clocks achieved robust prediction performance, although slightly diminished in comparison to chronological aging clocks (*R* = 0.41–0.89, error = 2.3–4.6 months; Fig. 1j,l). The predicted biological age was positively correlated with the predicted chronological age for each mouse (*R* = 0.84; Fig. 1h). Even though all biological aging clocks were trained on aNSC-NPC ‘proliferative fraction’, the microglia and oligodendrocytes biological age clocks performed better than aNSC-NPC ones (Fig. 1j,l).

Collectively, these data reveal that single-cell transcriptomes can be used to build accurate chronological and biological aging clocks for different cell types.

### External validation and generalization of aging clocks

**Fig. 2:**
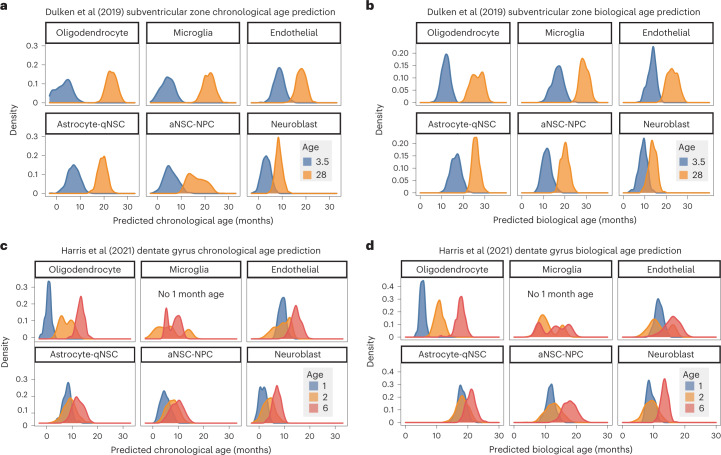
Generalization of aging clocks to independent datasets and other neurogenic regions. **a**, External validation of BootstrapCell chronological age prediction models (chronological aging clocks) on single-cell transcriptomic data from young (blue) and old (orange) SVZ samples by Dulken et al.^48^. Density plots show separated age prediction distributions, indicating ability to discriminate age. **b**, As in **a** but evaluating biological age prediction models (biological aging clocks). **c**, Density plots to assess the generalizability of chronological aging clocks (BootstrapCell) to another mouse neurogenic region using a dataset from Harris et al.^60^. Transcriptomes of analogous cell types were collected from the dentate gyrus of the hippocampus (another neurogenic region) instead of the SVZ in mice of different ages. There were no microglia in the dataset at the 1-month time point. **d**, Density plots to assess the generalizability of biological aging clocks (BootstrapCell) to another mouse neurogenic region using a dataset from Harris et al.^60^ as in **c**.

To externally validate these cell-specific aging clocks, we retrained chronological and biological aging clocks on all 28 mice and applied them to independent datasets. Our single-cell-based models easily separated young and old samples in an independent single-cell RNA-seq dataset from SVZ neurogenic regions of young and old mice^48^ (Fig. 2a,b). All cell-type-specific clocks effectively separated young and old samples, although the exact month of predicted age was more accurate with chronological clocks from some cell types (for example, microglia) compared to others (for example, neuroblasts; Fig. 2a,b).The successful application of our aging clocks to these independent datasets demonstrates their robustness.

We next asked if chronological and biological aging clocks could generalize to the same cell types in other regions of the brains and in other species. We first used a publicly available single-cell RNA-seq dataset with the same cell types from the other neurogenic region in the brain—the dentate gyrus of the hippocampus—in mice of different ages^60^. Our aging clocks properly separated samples of different ages in the dentate gyrus of the hippocampus, even samples only 1 month apart in age (Fig. 2c,d). We then used a publicly available single-nucleus RNA-seq datasets with oligodendrocytes and astrocytes from the middle temporal gyrus of the brain of humans of different ages^61^. Our chronological aging clocks could predict ages from both cell types, and these ages correlated with the actual ages of the humans (*R* = 0.75 for oligodendrocytes; *R* = 0.43 for astrocytes; Extended Data Fig. 1e). Thus, cell-type-specific aging clocks derived from mouse SVZ neurogenic regions generalize to the same cell types in other regions of the brain and to other species, including humans.

We also determined if the approach we used to build cell-type-specific chronological aging clocks was generalizable to other cell types in tissues other than the brain. Chronological aging clocks generated from endothelial cells from limb muscle, natural killer T cells from spleen and podocytes from kidney, using single-cell RNA-seq data from the multi-tissue aging atlas Tabula Muris Senis^62^ also exhibited great performance (*R* values ranging from 0.94 to 0.98) to predict actual chronological age (Extended Data Fig. 1f). Hence, cell-type-specific aging clocks can be derived from single-cell transcriptomics datasets from various cell types and tissues.

### Genes that contribute to the cell-type-specific aging clocks

**Fig. 3:**
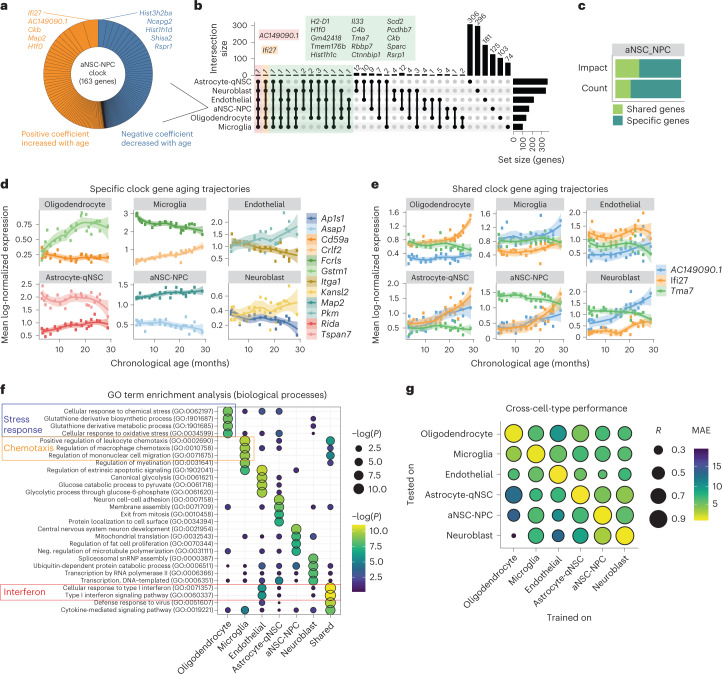
Genes underlying the cell-type-specific chronological aging clocks. **a**, Contribution of individual genes to the aNSC-NPC chronological aging clock (BootstrapCell). Donut plots, with sector size denoting gene weight in the model and color indicating sign of expression change with age. Total number of genes used by the clock is in the center. Positive coefficients (orange) indicate increased gene expression in older age. Negative coefficients (blue) indicate decreased gene expression in older age. For other chronological and biological aging clocks, see Extended Data Fig. 2a,b. All genes and coefficients are in Supplementary Table 3. **b**, UpSet plot illustrating the intersection of gene sets used by cell-type-specific chronological aging clocks. Genes present in four, five or six of the analyzed clocks are highlighted in green, yellow or red, respectively. For biological aging clocks, see Extended Data Fig. 2c. **c**, Bar plot comparing the impact and count of shared and specific genes within the aNSC-NPC chronological aging clock. Impact is the sum of the absolute value of the gene coefficients. Count is the number of genes in each category. For other chronological and biological aging clocks, see Extended Data Fig. 2d,e. **d**, Expression trajectories as a function of age of select clock-specific genes. Expression values are log-normalized counts per 10,000 transcripts. Bands correspond to 95% confidence intervals. **e**, Expression trajectories as a function of age of select shared genes across at least four cell-type-specific clocks. Bands correspond to 95% confidence intervals. **f**, Top enriched Gene Ontology (GO) biological process terms from GSEA of genes selected by chronological aging clocks. **g**, Assessment of the ability of cell-type-specific clocks to predict age given transcriptomes of different cell types. Size of dots corresponds to Pearson correlation with chronological age and color indicates MAE. Error substantially increases when testing on alternate cell types. For GO term analysis of cell-specific biological aging clocks, see Extended Data Fig. 2f.

**Extended Data Fig. 2:**
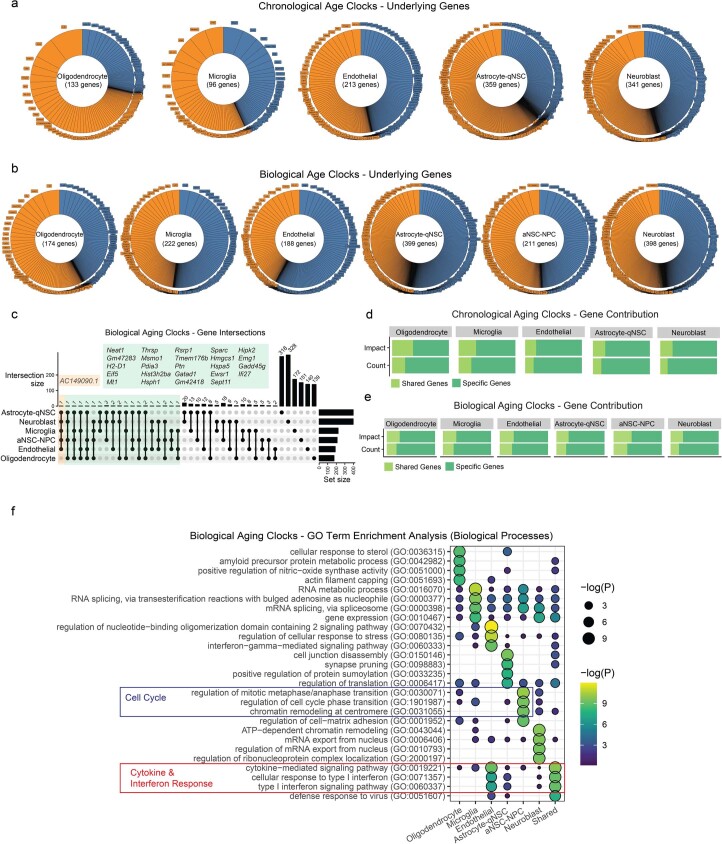
Genes that contribute to the chronological aging clocks and biological aging clocks. **a**, Contribution of individual genes to the chronological aging clocks (BootstrapCell) (see Fig. 3a for aNSC-NPCs). Donut plots, with sector size denoting gene weight in the model and color indicating sign of expression change with age. Total number of genes used by the clock is provided in the center of each donut plot. Positive coefficients (orange) indicate increased gene expression is associated with older age. Negative coefficients (blue) indicate decreased gene expression is associated with older age. **b**, As in (a) but for biological aging clocks (BootstrapCell) and their coefficients. **c**, Upset plot illustrating the intersection of gene sets used by cell-type-specific biological aging clocks. No genes were used in all 6 biological aging clocks. **d**, Count and coefficient impact of shared and cell-type-specific clock genes for chronological aging clocks. Shared is defined as present in at least one of the other five clocks (see Fig. 3c for aNSC-NPCs). **e**, As in (d) but for biological aging clocks. **f**, Top enriched Gene Ontology Biological Process terms from gene set enrichment analysis of genes used in biological aging clocks. Shared genes (present in two or more clocks) are enriched for *cytokine-mediated signaling pathway* and *cellular response to type I interferon*. The aNSC-NPC biological aging clock genes are enriched for cell cycle pathways.

**Extended Data Fig. 3:**
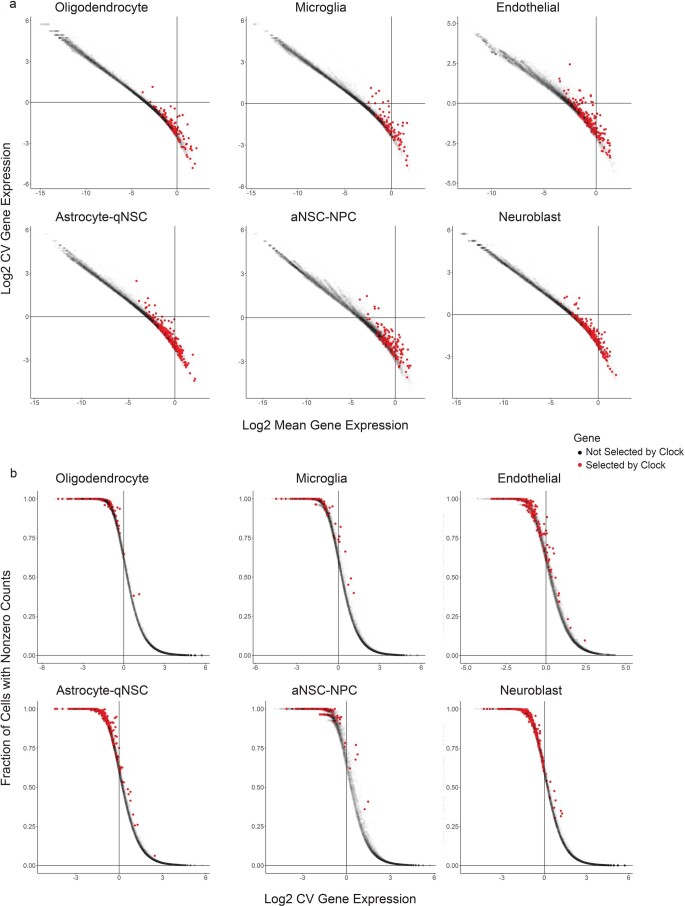
Variability and mean expression of genes in the chronological aging clocks. **a**, Scatter plots of the log2 coefficient of variation (CV) of the normalized BootstrapCell gene expression as a function of the log2 mean normalized BootstrapCell gene expression for all identified genes in the six different cell types. Red dots correspond to genes that contribute to the chronological aging clocks for each cell type (selected by clock) and black dots correspond to genes that do not contribute to the chronological aging clocks (not selected by clock). **b**, As in (a) but for the fraction of cells with nonzero counts in the dataset as a function of the log2 coefficient of variation (CV) of the normalized gene expression.

**Extended Data Fig. 4:**
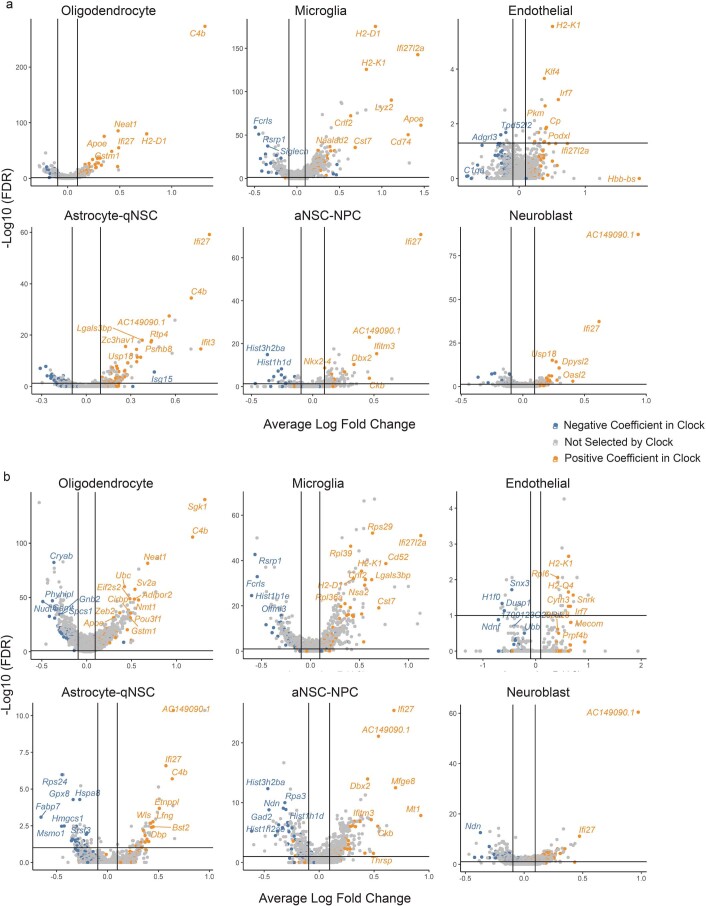
Comparison of genes in cell-type-specific aging clocks and differentially expressed genes with age. **a**, Volcano plots of negative log10 false discovery rate (FDR) from differential gene expression analysis using MAST for the young and old mouse groups. We defined ‘young’ as mice <7 months old and ‘old’ as mice >20 months old. Colored dots correspond to genes that contribute to the chronological aging clocks for each cell type (selected by clock) (orange representing genes with positive clock coefficients and blue representing genes with negative clock coefficients) and gray dots correspond to that do not contribute to the chronological aging clocks (not selected by clock). **b**, As in (a) but for the biological aging clocks.

What makes these aging clocks ‘tick’—that is, what are the genes that contribute to the aging clocks in each cell type? In the process of training, each clock selects top genes useful for accurate prediction and weighs the importance of each. To analyze selected genes and their relative contributions, we visualized each chronological or biological aging clock as a donut plot with genes that contribute the most at the top (Fig. 3a and Extended Data Fig. 2a,b). Gene sets contributing to the chronological and biological aging clocks in different cell types ranging from 96 to 359 genes (for chronological clocks) and 174 to 399 genes (for biological clocks), and they encompassed genes whose expression generally increased or decreased with age (Fig. 3a, Extended Data Fig. 2a,b and Supplementary Table 4). Genes selected by the aging clocks generally had higher mean expression and greater variability between mice (relative to expression level) compared to other genes (Extended Data Fig. 3a,b) and were differentially expressed during aging (Extended Data Fig. 4a,b).

The top genes contributing to the aNSC-NPC chronological aging clock were AC149090.1 and *Ifi27*, which are both upregulated with age (Fig. 3a). AC149090.1 is orthologous to human *PISD*, a gene encoding a phospholipid decarboxylase involved in lipid metabolism (phosphatidylethanolamine production), linked to autophagy, and localized to the inner mitochondrial membrane^63,64^. *Ifi27* (also referred to as *Isg12)* is a transcript upregulated in response to type I interferons^65^ (Fig. 3a). Thus, aging clocks identify many genes, including inflammation and lipid metabolism genes, whose expression is most predictive of aging in a particular cell type.

**Extended Data Fig. 5:**
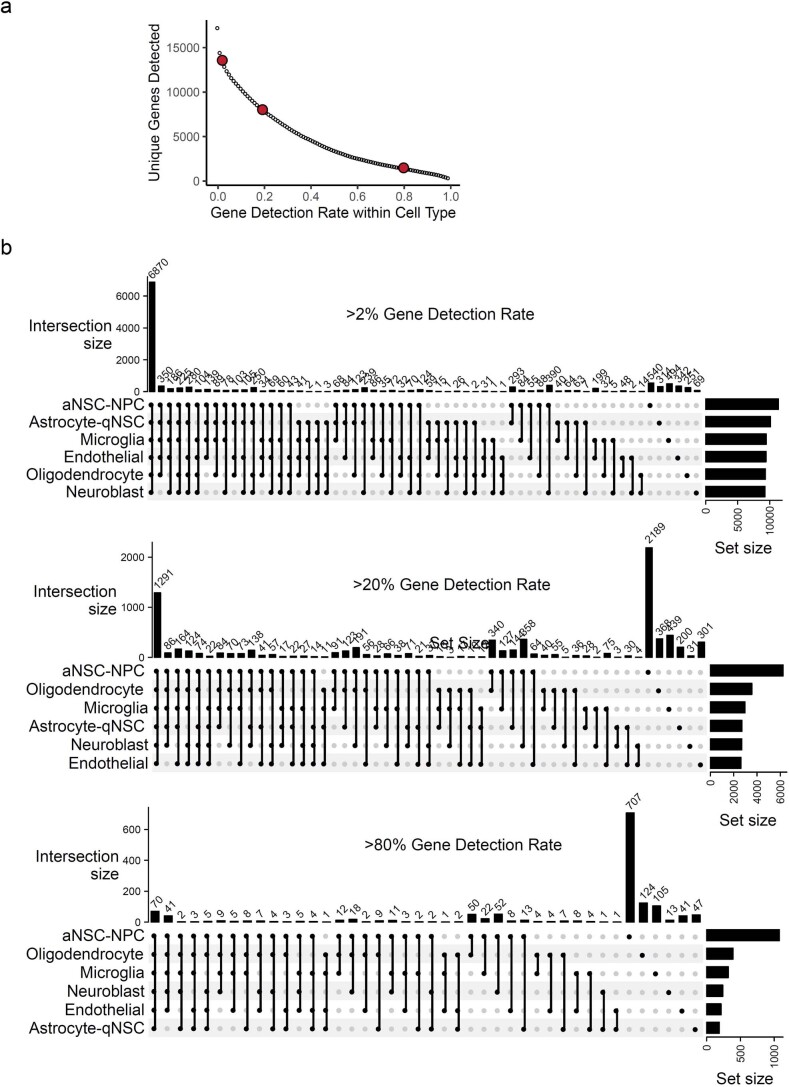
Gene detection rate in cell-type-specific aging clocks. **a**, Unique genes detected in single cell transcriptomes from the subventricular zone as a function of gene detection rate. Red dots indicate unique genes detected at 2%, 20%, and 80% detection rates. **b**, Upset plots showing transcriptome overlaps between cell types at different levels of expression detection. Most genes are shared if a very low threshold of detection is used. Above 80% detection rate, transcriptomes are very cell type specific. However, the shared core of easily detected genes in transcriptomes (70 genes) is much larger than the shared core of genes selected by clocks (~1).

To investigate whether each cell-type-specific clock selects similar or unique genes, we compared intersections of chronological or biological aging clock gene sets (Fig. 3b and Extended Data Fig. 2c). Interestingly, AC149090.1, was selected by chronological aging clocks from all six different cell types (Fig. 3b) and *Ifi27* was selected by chronological aging clocks from five of six cell types (Fig. 3b). In contrast, most genes selected by the cell-type-specific clocks were cell-type specific (Fig. 3b and Extended Data Fig. 2c). The cell-type specificity of the clocks exceeded what would be expected from transcriptome cell-type specificity alone (Extended Data Fig. 5a,b). However, shared selected genes carry a disproportionate weight within the clocks, with coefficients approximately 40% larger in magnitude (Fig. 3c and Extended Data Fig. 2d,e). Cell-type-specific genes (Fig. 3d) and even shared genes (Fig. 3e) exhibited differences in trajectory shapes (*Fcrls* and *Crlf2* in microglia) and expression magnitudes (for example, *Ifi27* in different cell types from the NSC lineage) during aging in different cell types. Thus, cell-type-specific clocks capture useful cell-type-specific expression differences and dynamics that would be missed by bulk methods.

While genes selected by the aging clocks are mostly cell-type specific, the pathways to which they belong could still be widely shared across cell types. To test this possibility, we examined the pathways enriched in the specific or shared genes selected by the chronological aging clocks. Interestingly, gene-set enrichment analysis (GSEA) on the specific genes from each chronological or biological clock revealed enrichment for different biological processes in each cell type (Fig. 3f and Extended Data Fig. 2f), for example, stress response for oligodendrocytes and chemotaxis for microglia (Fig. 3f). Thus, pathways for specific genes selected by the clocks are also largely cell-type specific and may reflect age-dependent changes in function in each cell type. There were a few biological processes most enriched in shared genes, including response to type I interferon and cytokine signaling (Fig. 3f and Extended Data Fig. 2f). Hence, our dissection of the genes composing the clocks highlights specific and common features of cellular aging, including stress response, lipid metabolism and inflammation.

Cell-type-specific clocks generated in one cell type did not perform as well on a different cell type (Fig. 3g), even though there are shared genes across all clocks and these are more heavily weighted. Thus, generating an aging clock from a specific cell type is helpful for accurately predicting the age of an individual from that cell type (Extended Data Fig. 1f).

Together, these data indicate that single-cell-based clocks select highly cell-type-specific genes to predict the age of the individual they come from, suggesting different aging trajectories in distinct cell types.

### Aging clocks capture the rejuvenating effect of parabiosis

**Fig. 4:**
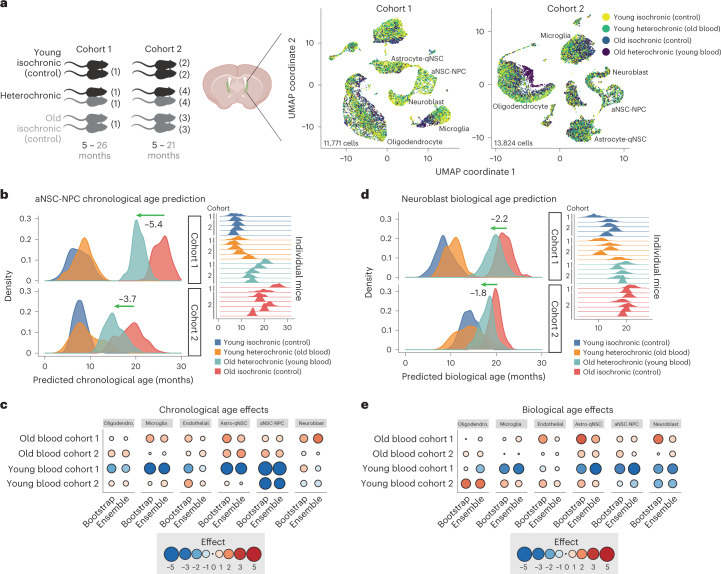
Effect of heterochronic parabiosis on cell-type-specific aging clocks. **a**, Schematic of parabiosis cohorts and corresponding UMAP projections from each cohort. Parabiosis cohort 1 dataset was generated with young (5 months) and old (26 months) male mice (number of mice indicated in parentheses); 11,771 high-quality transcriptomes were collected, using one SVZ sample per 10x lane. Parabiosis cohort 2 was generated with young (5 months) and old (21 months) male mice; 13,824 high-quality transcriptomes were collected, using LMOs to multiplex SVZ samples across three 10x lanes. UMAP projection and cell type clustering of SVZ single-cell transcriptomes in cohorts 1 and 2. Each dot represents the transcriptome of an individual cell. Colored by age and intervention (heterochronic parabiosis). For coloration by cell type, see Extended Data Fig. 6a. **b**, Density plots of the predicted chronological ages for aNSC-NPCs from cohort 1 and cohort 2. Green arrows illustrate the median shift in predicted age between old aNSC-NPCs exposed to young blood (old heterochronic) and old aNSC-NPCs exposed to old blood (old isochronic, control). Density plots for individual mice, and their cohorts of origin, are provided on the right. **c**, Summary of heterochronic parabiosis effects on chronological age scores across cell types. Effect sizes were calculated by taking the difference in median predicted ages between conditions. Blue color indicates a decrease in predicted chronological age (‘rejuvenation’). Red color indicates an increase in predicted chronological age (‘detrimental impact’). **d**, Density plots of the predicted biological age scores for neuroblasts from cohort 1 and cohort 2. Green arrows illustrate the median shift in predicted age between old neuroblasts exposed to young circulation (old heterochronic) compared to old neuroblasts exposed to old circulation (old isochronic, control). Density plots for individual mice, and their cohort of origin, are provided on the right. **e**, Summary of heterochronic parabiosis effects on biological age scores across cell types. Effect sizes are calculated by taking the difference in median predicted ages between conditions. Blue indicates a decrease in predicted chronological age (‘rejuvenation’). Red indicates an increase in predicted biological age. For statistical analysis at the mouse level, see Extended Data Fig. 7a.

**Extended Data Fig. 6:**
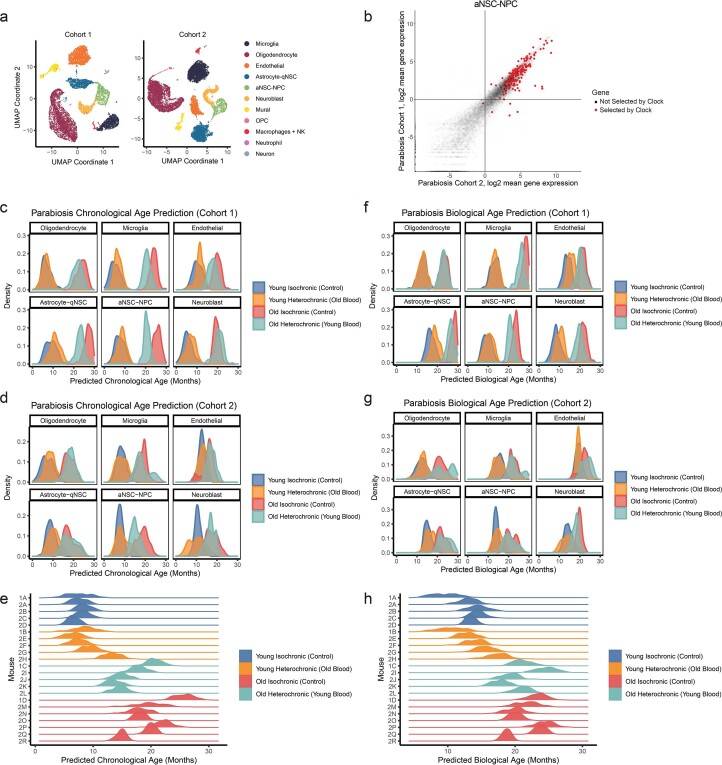
Application of cell-type-specific aging clocks to heterochronic parabiosis cohorts. **a**, UMAP projection of single-cell transcriptomes labeled by cell type from both parabiosis cohorts. **b**, Scatter plot of log2 mean BootstrapCell expression of all genes in the parabiosis cohort 1 data compared to the same genes in the parabiosis cohort 2 data for aNSC-NPCs. Red dots correspond to genes that contribute to the aNSC-NPC chronological aging clock (selected by clock). Gray dots correspond to genes that do not contribute to the aNSC-NPC chronological aging clock (not selected by clock). **c**, Density plots for prediction of chronological age using chronological aging clocks for Parabiosis cohort 1 (BootstrapCell). **d**, Density plots for prediction of chronological age using chronological aging clocks for Parabiosis cohort 2 (BoostrapCell). **e**, Density plots for prediction of chronological age using chronological aging clocks for both cohorts separated by mouse (BootstrapCell). **f**, Density plots for prediction of biological age using biological aging clocks for Parabiosis cohort 1 (BootstrapCell). **g**, Density plots for prediction of biological age using biological aging clocks for Parabiosis cohort 2 (BoostrapCell). **h**, Density plots for prediction of biological age using biological aging clocks for both cohorts separated by mouse (BoostrapCell).

Do single-cell-based aging clocks—whether trained on chronological or biological age—capture known ‘rejuvenating’ interventions? A robust rejuvenating intervention across tissues is heterochronic parabiosis—the sharing of blood circulation between young and old animals^66–77^. Parabiosis with a young animal can restore aspects of cell function (for example, NSC proliferation and vascular remodeling) in neurogenic regions of an old animal, and part of the effects can be recapitulated by the injection of young blood or plasma^69,78,79^. To test how our single-cell-based aging clocks recorded the impact of exposure to young and old blood on neurogenic regions, we generated multiplexed single-cell RNA-seq data on SVZ neurogenic regions from heterochronic parabiosed young and old mice and isochronic parabiosed controls. In total, we collected 25,595 single-cell transcriptomes from the SVZ neurogenic regions of 22 mice across 2 independent cohorts (Fig. 4a, Extended Data Fig. 6a,b, Supplementary Table 1 and Methods). Mean gene expression was similar in both cohorts for most clock genes (Extended Data Fig. 6b). This dataset represents a single-cell RNA-seq resource for heterochronic parabiosis in the SVZ neurogenic region.

**Extended Data Fig. 7:**
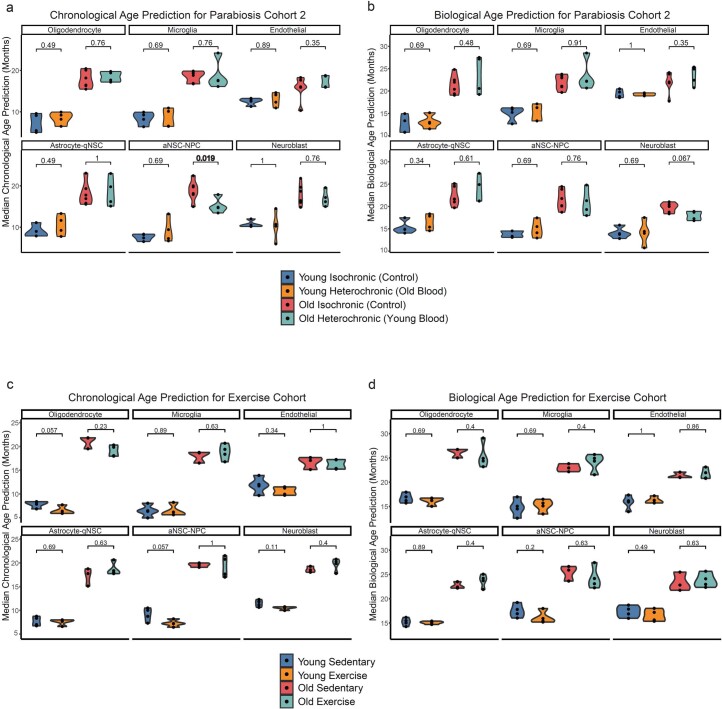
Statistical comparison of the rejuvenation effect size for heterochronic parabiosis and exercise at the mouse level. **a**, Violin plots of the median predicted chronological ages for all mice in parabiosis cohort 2 (BootstrapCell). Each dot correspond to the median predicted chronological age of an individual mouse. P-values at the mouse level obtained from the two-sided Wilcoxon rank-sum test. **b**, As in (a) but for the median predicted biological ages. **c**, As in (a) but for the exercise cohort. **d**, As in (a) but for the median predicted biological age in the exercise cohort.

Applying our suite of cell-type-specific aging clocks, we predicted both the chronological and biological ages of individuals in response to heterochronic parabiosis. Interestingly, mice exposed to young blood showed a striking rejuvenation effect in aNSC-NPCs, across both cohorts for chronological age (rejuvenation of 5.38 months in cohort 1 and 3.66 months in cohort 2; 4.52 months, averaging both cohorts; Fig. 4b,c and Extended Data Fig. 6c–e) and to a lesser extent for biological age (rejuvenation of 3.57 months in cohort 1 and 1.44 months in cohort 2; 2.51 months, averaging both cohorts; Fig. 4d,e and Extended Data Fig. 6f–h). The effect of young blood was statistically significant at the mouse level (*P* = 0.019) in aNSC-NPCs, for chronological aging clocks in cohort 2 (which had 4–6 mice per condition; Extended Data Fig. 7a,b). In other cell types, there was a tendency toward a rejuvenation effect, particularly in microglia and neuroblasts (although with less consistency between cohorts; Fig. 4c,e and Extended Data Figs. 6c–h and 7a,b). Overall, the first cohort (21 months difference between young and old) showed a stronger rejuvenation effect than the second cohort (15.5 months difference between young and old; Fig. 4c,e), suggesting an improved effect of exposure to young blood on older animals (or a greater magnitude when the difference in age in parabionts is larger). There was no correlation between rejuvenation effect size and clock performance, suggesting that differences in intervention effects across cell types was not primarily due to differences in the performance of their respective cell-type-specific aging clocks. Conversely, aging clocks also revealed that young mice exposed to old blood experienced an increase of predicted chronological age across several cell types (Fig. 4c,e), confirming the detrimental impact of old blood in other tissues^74,77,80–84^.

Thus, single-cell-based aging clocks, even when trained on chronological age, can be used to quantify the impact and magnitude of rejuvenation and pro-aging interventions on different cell types. Furthermore, these clocks uncover cell-specific rejuvenating effects in old neurogenic regions exposed to young blood focused on proliferating NSCs.

### Aging clocks capture the rejuvenating effect of exercise

**Fig. 5:**
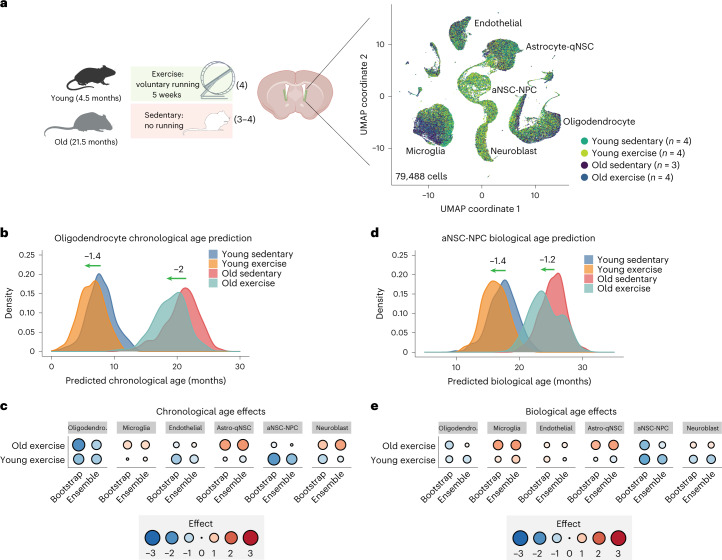
Effect of exercise on cell-type-specific aging clocks. **a**, Schematic of voluntary wheel running experiment and UMAP projection of single-cell transcriptomes. For the exercise cohort, 4 young (4.5 months) or 3–4 old (21.5 months) male mice were transferred into cages with either a freely spinning wheel or no wheel. Wheel rotations were tracked to verify that mice indeed exercised. After 5 weeks, SVZ niches were collected, so mice were ~6 months and 23 months at time of collection, and 15 lanes of 10x Genomics transcriptomics performed without sample multiplexing. UMAP projection and cell-type clustering of SVZ single-cell transcriptomes in the exercise cohort. Each dot represents the transcriptome of an individual cell. Colored by age and intervention (exercise) or by cell type (UMAP; Extended Data Fig. 8a). **b**, Density plots of predicted chronological ages of oligodendrocytes by age and exercise condition. Exercise consistently rejuvenated oligodendrocyte transcriptomes regardless of age. **c**, Summary of exercise effects on chronological age scores across cell types and ages. Effect sizes were calculated by taking the difference in median predicted ages between conditions. Blue indicates a decrease in predicted chronological age (‘rejuvenation’). Red indicates an increase in predicted chronological age (‘detrimental impact’). **d**, Density plots of aNSC-NPC predicted biological ages. Exercise rejuvenated aNSC-NPC transcriptomes of both young and old mice. **e**, Summary of exercise effects on biological age scores across cell types and ages. Effect sizes were calculated by taking the difference in median predicted ages between conditions. Blue indicates a decrease in predicted biological age (‘rejuvenation’). Red indicates an increase in predicted chronological age (‘detrimental impact’). For statistical analysis at the mouse level, see Extended Data Fig. 7b.

**Extended Data Fig. 8:**
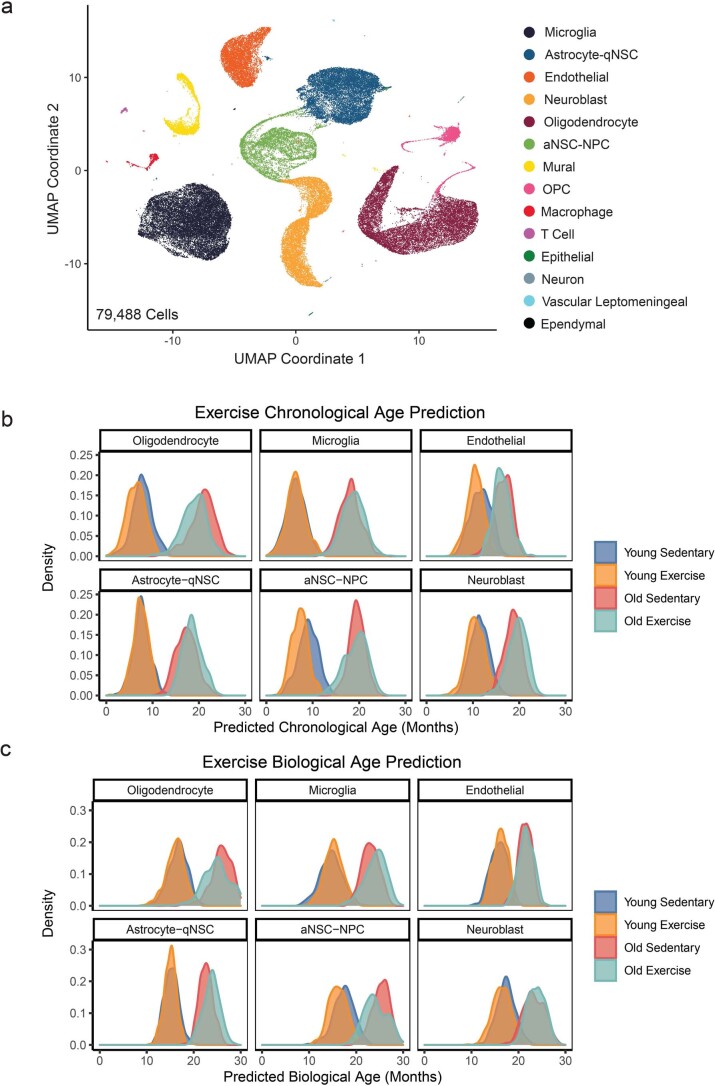
Application of cell-type-specific aging clocks to exercise cohort. **a**, UMAP projection of single-cell transcriptomes labeled by cell type from the exercise intervention cohort. **b**, Density plots to predict chronological age using chronological aging clocks (BootstrapCell) for young and old, exercise and control samples. **c**, Density plots to predict biological age using biological aging clocks (BoostrapCell) for young and old, exercise and control samples.

We asked if other interventions that are beneficial for health also had a ‘rejuvenating’ effect on cell-type-specific aging clocks. Thus, we applied clocks to another systemic intervention—exercise. Exercise via voluntary wheel running has beneficial effects on the brain, increasing hippocampal neurogenesis and improving memory^85–91^, and boosting SVZ neurogenesis in several cases^92–95^. We exercised young (4.5 months) and old (21.5 months) mice by providing 5 weeks of access to freely spinning wheels (or no wheels as controls) and verified that mice with this paradigm exercised (Fig. 5a, Extended Data Fig. 8a and Supplementary Table 1). We then generated 79,488 single-cell transcriptomes from the SVZ neurogenic region from young and old, exercised and non-exercised controls—a total of 15 mice (Fig. 5a and Methods). These single-cell RNA-seq data represent a great resource for the young and old SVZ neurogenic niche response to exercise.

Applying our chronological aging clocks to the exercise transcriptome dataset revealed that exercise had a small rejuvenation effect in oligodendrocytes (1.4 months in young, 2.0 months in old, not significant at the mouse level; Fig. 5b,c) and in aNSC-NPCs (1.9 months in young, 0.3 months in old; Extended Data Fig. 8b). Biological aging clocks also captured a small rejuvenation effect in oligodendrocytes and aNSC-NPCs (0.6 months in young, 0.8 months in old; and 1.4 months in young, 1.2 months in old, respectively; Fig. 5d,e and Extended Data Fig. 8c). The effect of exercise in young mice was trending at the mouse level (*P* = 0.057) in oligodendrocytes for chronological aging clocks (Extended Data Fig. 7c,d). Hence, single-cell-based aging clocks also identify rejuvenating trends for exercise in neurogenic regions, notably in aNSC-NPCs and oligodendrocytes.

### Comparison between heterochronic parabiosis and exercise

**Fig. 6:**
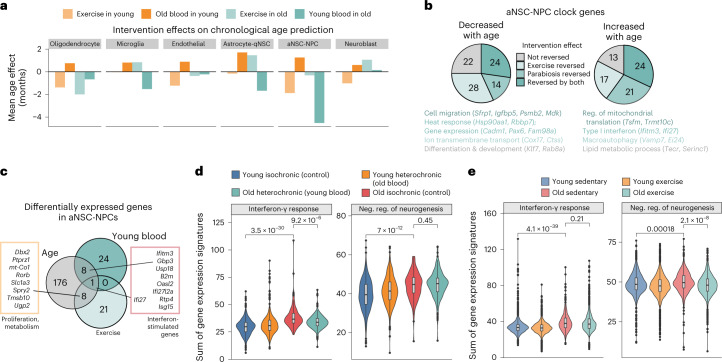
Comparison of exercise and parabiosis interventions on cell-type-specific aging clocks. **a**, Bar plot comparing effects of different interventions. Bar represents the difference (in months) between predicted chronological ages between controls and intervention. Parabiosis cohorts 1 and 2 were averaged. **b**, Pie charts of the directional effect and overlap of intervention impact on aNSC-NPC chronological aging clock genes (BootstrapCell). Genes are called ‘reversed’ when the sign of the log fold change of gene expression in intervention versus control is opposite to the sign of the coefficient of the gene in the clock (indicated on top of the pie charts). Top GO biological process terms and representative genes are listed underneath. **c**, Venn diagram representing the overlap of DEGs in aNSC-NPCs between young and old mice (‘age’), old heterochronic mice and old isochronic mice (‘young blood’) and old exercised and old sedentary mice (‘exercise’). Differential expression thresholds required a minimum 1.1-fold expression change with a false discovery rate (FDR) < 0.1. For aging, mice were grouped as either young (<7 months) or old (>20 months). DEGs shared between age and young blood were interferon-stimulated genes. DEGs shared between age and exercise were genes involved in proliferation, metabolism and development. **d**, Violin and box plots of gene signatures (sum of normalized gene expression for all genes in the gene set) for ‘interferon-γ response’ and ‘negative regulation of neurogenesis’ for aNSC-NPCs in the parabiosis cohort 1 and cohort 2 combined. In the box plot, the line represents the median and the box represents the interquartile range. *P* values were obtained from the two-sided Wilcoxon rank-sum test (*n* = 668, 149 and 146 cells for ‘young isochronic’, ‘old isochronic’ and ‘old heterochronic’, respectively). **e**, As in **d** but for aNSC-NPCs in the exercise cohort (*n* = 2,243, 503 and 1,170 cells for ‘young sedentary’, ‘old sedentary’ and ‘old exercise’, respectively).

**Extended Data Fig. 9:**
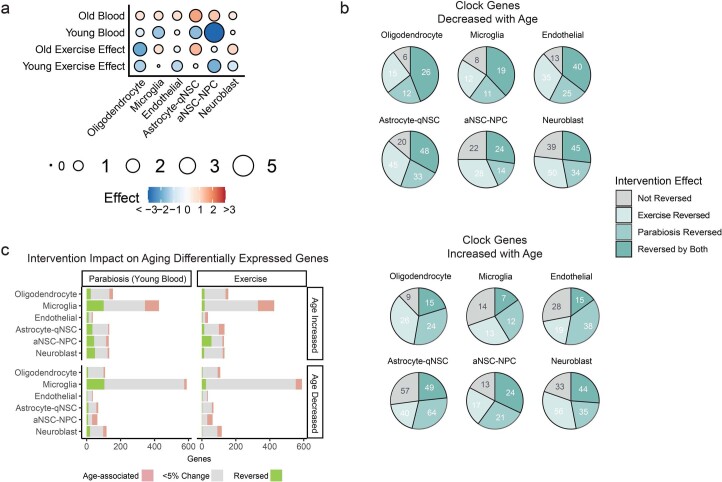
Comparison of genes in cell-type-specific aging clocks impacted by heterochronic parabiosis and by exercise. **a**, Dot plot summarizing and comparing intervention effects across cell types. Effect sizes for parabiosis were determined by averaging cohort 1 and cohort 2. Exposure to young blood via heterochronic parabiosis has a stronger rejuvenation effect than exercise, and the impact is strongest in aNSC-NPCs. **b**, Pie charts indicating the overlap and directional effects of different interventions on genes selected by chronological aging clocks (BootstrapCell). Top: selected clock genes increase with age: Bottom: selected clock genes decrease with age. **c**, Barplots showing the proportion of genes that are differentially expressed age which are reversed by intervention, cell type, and whether the genes increase or decrease with age (abs(ln(fold change)) > 0.1, or approximately greater than a 1.1 fold change with age, FDR < 0.1). Parabiosis is effective at shifting differentially expressed genes during aging towards a more young-associated expression levels (more green in ‘Parabiosis’ column). Reduction of expression of genes that increase with age is larger than the induction of expression of genes that decrease with age (more green in ‘Age Increased’ rows).

We compared the effect of heterochronic parabiosis and exercise on cell-type-specific aging clocks. Overall, heterochronic parabiosis (merging cohorts 1 and 2) had a larger rejuvenating effect than exercise across cell types (Fig. 6a). Young blood had a strong rejuvenating effect on old mice in aNSC-NPCs and had a smaller rejuvenating effect on microglia and neuroblasts (Figs. 4c,e and 6a and Extended Data Figs. 7a,b and 9a). Exercise also had a small rejuvenating effect on aNSC-NPCs and oligodendrocytes (Fig. 6a and Extended Data Figs. 7c,d and 9a). Together, these results suggest that exposure to young blood may be a stronger intervention than exercise, at least at the transcriptomic level, and may impact both shared (aNSC-NPCs) and distinct cell types.

We next examined the genes responding to either or both of these interventions in a cell type (aNSC-NPCs) responding to both interventions, albeit with a different magnitude. In aNSC-NPCs, heterochronic parabiosis mostly reversed clock genes that increased in expression with aging (such as those associated with a type I interferon response; Fig. 6b and Extended Data Fig. 9b). In contrast, exercise mostly reversed clock genes that decreased in expression with aging (such as those associated with transmembrane transport; Fig. 6b and Extended Data Fig. 9b). These results suggest that young blood and exercise target different genes and pathways.

**Extended Data Fig. 10:**
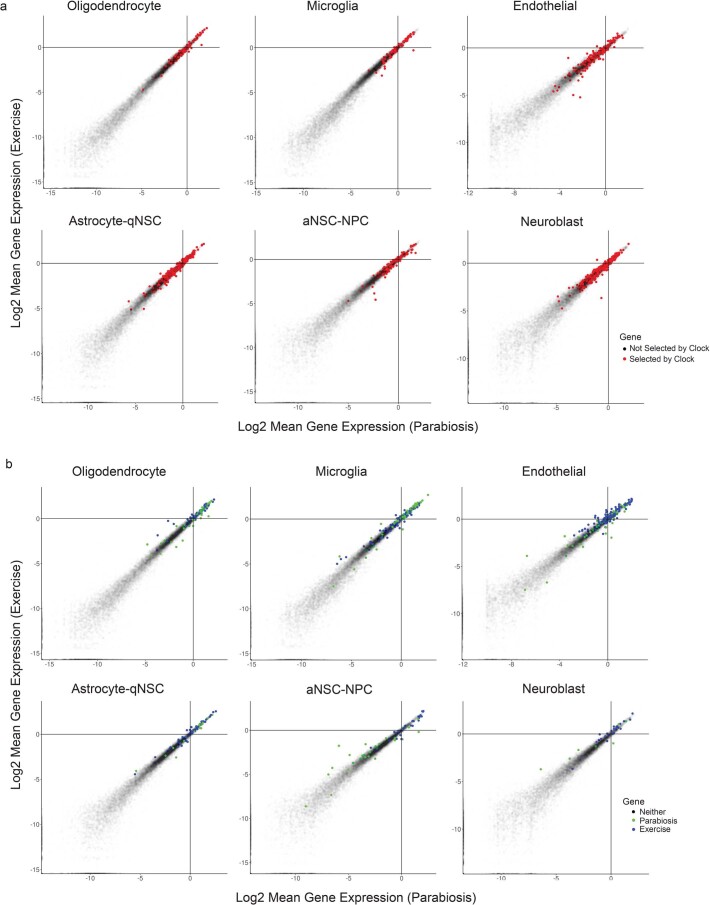
Comparison of mean expression of genes in the aging clocks and genes impacted by rejuvenation in the heterochronic parabiosis and exercise datasets. **a**, Scatter plots of the log2 mean normalized BootstrapCell gene expression in the exercise single-cell data as a function of the log2 mean normalized BootstrapCell gene expression in the parabiosis (cohorts 1 and 2 combined) single-cell data for the six main cell types. Red dots correspond to genes that contribute to the chronological aging clocks (selected by clock) and gray dots correspond to genes that do not contribute to the chronological aging clocks (not selected by clock). Genes that contribute to the clock are both highly expressed in the parabiosis and exercise datasets. **b**, As in (a) but colored dots correspond to genes identified as differentially expressed by parabiosis (green) and exercise (blue). Gray dots correspond to genes that are affected neither by parabiosis nor by exercise (neither). Permissive 1.1-fold change and FDR < 0.1 cutoffs were applied in each of the 2 different conditions.

To independently test whether heterochronic parabiosis and exercise impact different genes, we examined differentially expressed genes (DEGs) in aNSC-NPCs during aging and in response to each intervention (Methods and Fig. 6c). There was minimal overlap between parabiosis-responsive and exercise-responsive genes (by DEG), corroborating that these interventions impact the aging transcriptome differently (Fig. 6c). This minimal overlap was not due to a drastically different overall pattern of gene expression between the parabiosis and exercise datasets (Extended Data Fig. 10). Young blood reversed the age-associated increase in interferon-stimulated genes (including the shared gene *Ifi27*; Fig. 6c). In contrast, exercise reversed the age-associated decline of several genes involved in proliferation and neurogenesis, including *Dbx2*, which is implicated in age-related SVZ neurogenic decline^96^ (Fig. 6c). Young blood but not exercise reversed the age-associated increase in genes involved in the ‘interferon-γ response’ signature (Fig. 6d). Exercise but not young blood reversed the age-associated decrease in the ‘negative regulation of neurogenesis’ signature (Fig. 6e), consistent with the ability of exercise to boost neurogenesis in old mice^88–91,97^. DEG analysis also confirmed that the impact of young blood on DEGs was stronger than that of exercise across most cell types (Extended Data Fig. 9c). Together, these data corroborate that the transcriptional responses of old neurogenic regions to heterochronic parabiosis and exercise differ.

### Relevance of rejuvenation interventions to aging

**Fig. 7:**
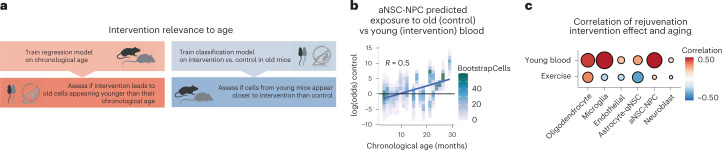
Predicting ‘rejuvenation intervention or control’ state on the transcriptomes from mice of different ages to assess intervention relevance to aging. **a**, Schematic describing how to predict ‘rejuvenation intervention or control’ state on the transcriptomes from mice of different ages to assess intervention relevance to aging. **b**, Classification results based on logistic regression for the parabiosis intervention in aNSC-NPCs. Correlation between classification results, plotted as (log(*p*(control) / *p*(intervention))) and the actual chronological age of aNSC-NPC BootstrapCell transcriptomes. Old mice were more likely to be classified as ‘isochronic old control’, whereas young mice were more likely to be classified as ‘heterochronic old’, indicating that the gene signature that distinguishes exposure to young and old blood is relevant to aging. *R* is the Pearson correlation. Higher correlation indicates that the main intervention signature overlaps with and reverses age-related changes. **c**, Summary of correlations between intervention state prediction and chronological age across cell types and interventions, with a separate classifier built for each. The exercise classifiers were built to distinguish old sedentary from old exercised transcriptomes for each cell type. The lower correlation between intervention state predictions and age for the exercise samples implies that the signatures that distinguish exercised and sedentary mice are less related to aging than those derived from parabiosis intervention classifiers.

Importantly, we determined whether the main effects of exposure to young blood and exercise are indeed relevant to aging (Fig. 7a). To this end, instead of training regression clocks on age to compare rejuvenation interventions, we trained classifiers on rejuvenation interventions (‘rejuvenated’ and ‘control’) and determined if different chronological ages were classified as rejuvenated or control. With the classifier built on heterochronic parabiosis, younger mice showed a greater likelihood of being classified as ‘rejuvenated’ whereas older mice showed a greater likelihood of being classified as ‘control’ (Fig. 7b). This effect was particularly strong in aNSC-NPCs and microglia (Fig. 7b,c). In contrast, with the classifier built on exercise, younger mice showed a greater likelihood of being classified as ‘rejuvenated’ in only two of six cell types (aNSC-NPCs and oligodendrocytes; Fig. 7b), which were also the same two cell types that showed the strongest rejuvenation from aging clock analysis (Fig. 6a). This analysis indicates that exercise and young blood induce changes that are indeed relevant to aging and corroborates the comparatively larger effects of young blood as an intervention. Collectively, these machine learning analyses have the potential to identify differences in rejuvenation interventions.

## Discussion

Here we show that single-cell RNA-seq data allow the generation of quantitative aging clocks that can be trained on chronological age or on aspect of tissue fitness (defined here as ‘biological age’)—that is, the proliferative fraction of stem cells in the neurogenic region. To our knowledge, these are the first quantitative aging clocks in distinct cell types based on single-cell RNA-seq. We also generate three datasets that represent valuable stand-alone resources: a high temporal resolution single-cell RNA-seq aging dataset of a neurogenic niche and single-cell RNA-seq datasets for a neurogenic niche following heterochronic parabiosis and voluntary exercise. These datasets will be helpful to identify additional cellular and molecular changes during aging and rejuvenation.

Our clock accuracy (for example, *R* = 0.92 in microglia) approaches that of bulk DNA methylation and proteomics^11,15,30^ while preserving cell-type specificity and avoiding biased sorting procedures. Single-cell DNA methylation and proteomic methods have suffered from sparsity and scaling challenges, although there is rapid innovation to address these issues^36,98^. While the methods described here preserve cell-type specificity without relying on cell sorting, ‘pure’ single-cell trained clocks were not as effective as our BootstrapCell and EnsembleCell approaches (Fig. 1k,l). Thus, using small pools of 15 single-cell transcriptomes can mitigate some technological (for example, gene dropouts) or biological (for example, transcriptional bursting) challenges inherent to single-cell RNA-seq datasets. Nevertheless, increased gene variability (transcriptional noise) is itself a feature of aging^99–103^, and it will be important to model this feature in the next-generation aging clocks.

A limitation of the application of chronological age-trained clocks is that interventions stimulating age-associated compensatory pathways (for example, stress responses) will reflect as age-acceleration interventions despite their functional benefit to the cell, the tissue or the organism. Thus, there is a need for a better understanding of genes contributing to the aging clocks and their function as well as continued development of functional and phenotypic-trained models. Here, we built clocks based on a functional phenotype of the neurogenic region (NSC proliferative capacity), but more comprehensive phenotyping approaches will be important to pursue. Overall, functional-aging clocks are likely to be instrumental in understanding the biology of aging and rapidly evaluating interventions necessary to extend healthy lifespan.

The observation that heterochronic parabiosis and exercise can ‘turn back’ the single-cell-based aging clocks provides a proof of concept that these aging clocks, even when trained on chronological age, can record aspects of aging biology. This is in line with other aging clocks built on bulk datasets^11,21–24,31,32^. Our results also highlight cell-type specificity for aging and possibly for rejuvenation interventions. This is unique to single-cell-based clocks and will allow a better understanding of cell heterogeneity in tissue aging and rejuvenation. Our data also reveal different potential for rejuvenation strategies, at least at the transcriptional level. These results raise the exciting possibility that aging clocks can serve to rapidly test the efficacy of rejuvenation interventions and to support combining specific interventions to counter aging and age-related diseases.

## Methods

Our research complies with all relevant ethical regulations (AAALAC), under Institutional Animal Care and Use (IACUC) protocols 8661 and 16246 at Stanford University and VA Palo Alto Committee on Animal Research, ACORP LUO1736.

### Animals

For aging cohorts and the exercise cohort, male C57BL/6 mice were obtained from the National Institute on Aging (NIA) Aged Rodent colony. For parabiosis cohort 1, old mice were male C57BL/6 mice from the NIA Aged Rodent colony and young mice were male B6.SJL-*Ptprc*^*a*^ *Pepc*^*b*^/BoyJ male (Pep boy) from the Jackson Laboratory. For parabiosis cohort 2, old mice were male C57BL/6J and young mice were male C57BL/6J or C57BL/6-Tg(UBC-GFP)30Scha/J from the Jackson Laboratory. Mice were housed in the Comparative Medicine Pavilion, ChemH/Neuroscience Vivarium or the SIM-1 Non-Barrier Rodent Facility at Stanford, or in the Veterinary Medical Unit at the Palo Alto VA. All these facilities provide equivalent standard conditions with a 12-h light–dark cycle, ad libitum food and water, ~21 °C temperature, and ~50% humidity. All mice were acclimated to their vivarium for at least 2 weeks before use in any experiment.

### Tissue and cell collection for the subventricular zone neurogenic niche

For single-cell RNA-seq datasets, SVZ neurogenic niches were collected and processed as described in ref. ^48^. Briefly, mice were sedated with 1 ml of 2.5% vol/vol Avertin (Sigma-Aldrich, T48402-25G) and perfused with 15 ml of PBS (Corning, 21-040-CV) with heparin sodium salt (50 U ml^−1^; Sigma-Aldrich, H3149-50KU) to remove the blood, and brain collection was performed immediately. As previously described^104^, the SVZ from each hemisphere was microdissected and dissociated with enzymatic digestion with papain at a concentration of 14 U ml^−1^, rocking for 10 min at 37 °C. Note that the samples also contained some of the surrounding striatum, which contributed to the oligodendrocyte population in our study. The dissociated SVZ was triturated in a solution containing 0.7 mg ml^−1^ ovomucoid and 0.5 mg ml^−1^ DNase I (Sigma-Aldrich, DN25-100MG) in DMEM/F12 (Thermo Fisher, 11330032). The dissociated cells from the SVZ were centrifuged through 22% Percoll (Sigma-Aldrich, GE17-0891-01) in PBS to remove myelin debris. After centrifugation, cells were filtered through a 35-μm snap-cap filter (Corning, 352235), washed once with 1.5 ml of FACS buffer (HBSS (Thermo Fisher, 14175103), 1% BSA (Sigma, A7979) and 0.1% glucose (Sigma-Aldrich, G7021-1KG)) and spun down for 5 min at 300*g*. Cells were resuspended in 120 μl FACS buffer with live/dead staining performed using 1 μg ml^−1^ propidium iodide (BioLegend, 421301) and kept on ice until sorting. FACS sorting was performed on a BD FACS Aria II sorter, using a 100-μm nozzle at 13.1 PSI. Cells were sorted into low protein binding microcentrifuge tubes containing 750 μl of PBS with 1% BSA and 0.1% glucose. When not applying sample multiplexing (parabiosis cohort 1 and exercise cohort), cells were then centrifuged (300*g* for 5 min at 4 °C) and resuspended in 50 μl FACS buffer, counted and then immediately run on 10x Chromium to capture single-cell transcriptomes.

### Cohorts of mice of different ages

To generate the single-cell RNA-seq dataset from mice of different ages and train aging clock models, we used four independent cohorts of aging mice. Each cohort had 4–8 male C57BL/6 mice from the NIA Aged Rodent colony, for a total of 28 mice. These 28 mice tiled 26 different ages (two pairs of mice had the same age), ranging from 3.3 months (young adult) to 29 months (geriatric adult).

### Lipid-modified oligonucleotide multiplexing

Sample multiplexing was performed using LMOs, a method also known as MULTI-seq^50^. Lipid anchor and co-anchor reagents were kindly provided by the Gartner Laboratory at the University of California, San Francisco and custom oligonucleotides were ordered from Integrated DNA Technologies. We used MULTI-seq primer: 5′ CTTGGCACCCGAGAATTCC; and Universal.I5: 5′AATGATACGGCGACCACCGAGATCTACACTCTTTCCCTACACGACGCTCTTCCGATCT^50^.

We followed the exact protocol outlined by McGinnis et al.^50^ with the following modifications: (1) all labeling with LMOs was performed in a 4 °C cold room because, in our hands, the quality of labeling was very sensitive to temperature; (2) to avoid cell loss and cell clumping, cells were sorted into PBS with 2% BSA, and BSA was then removed using three PBS washes; (3) concentrations and volumes were adjusted to account for low cell numbers: 7.5 μl of 1 mM lipid anchor with oligonucleotide barcode mix was added to a 70 μl volume of resuspended cells followed by 7.5 μl of 1 mM lipid co-anchor; (4) labeling reactions were quenched with 2% BSA then samples were pooled before subsequent 1% BSA PBS washes to further reduce cell loss. The combined sample was resuspended at 50 μl for cell counting and single-cell RNA-seq.

### Single-cell libraries and RNA sequencing

Single-cell RNA-seq was performed using a 10x Chromium machine and 10x Genomics V3.0 Transcriptomics kits (aging cohorts, parabiosis cohort 2 and exercise cohort) or a 10x Genomics V2 kit (parabiosis cohort 1). For sequencing, 10,000 cells per lane were targeted but typical yields were approximately 5,000 cells. Library preparation was done according to the manufacturer’s protocol (10x Genomics V3.0 or 10x Genomics V2 for parabiosis cohort 1). Sequencing was done to target a minimum of 25,000 reads per cell for transcriptome characterization and 5,000 reads per cell for LMO label recovery. The aging cohorts and the parabiosis cohort 2 samples were multiplexed with 4–8 samples per 10x Chromium lane. The parabiosis cohort 1 and the exercise samples were not multiplexed with LMO reagents. Sequencing was performed on either an Illumina HiSeq 4000 (aging cohorts and parabiosis cohort 1) or a NovoSeq using the 2 × 150-bp setting (parabiosis cohort 2 and exercise).

### Analysis (quality control)

Cell Ranger (version 3.0.2) default settings were used to distinguish cells from background. Subsequent analysis was performed using R (version 3.6.3). Cells were filtered out in Seurat (version 3.2.3)^105,106^ if they contained fewer than 500 genes or greater than 10% mitochondrial reads. Small clusters of doublets that shared several marker genes from pure populations were identified and removed. LMO demultiplexing was performed using Seurat’s HTODemux function. A complete view of the data processing and quality-control parameters can be found at https://github.com/sunericd/svz_singlecell_aging_clocks.

### Cell type annotation

Cell types in all datasets were manually annotated as described in ref. ^48^, and cross-referenced with annotations present in the single-cell database PanglaoDB^107^. Identification of major clusters was performed with the FindClusters() algorithm in the Seurat package, which uses a shared nearest-neighbor modularity optimization-based clustering algorithm^106^. Marker genes for each major cluster were found using the Seurat (version 4.1.1) function FindAllMarkers() using the Wilcoxon rank-sum test. Cell types were determined using marker genes identified from the literature and the marker genes were cross-referenced with annotations present in the single-cell database PanglaoDB^107^. This analysis identified ~11 clusters of cells (depending on the dataset), including astrocytes and qNSCs, aNSCs and NPCs, neuroblasts, neurons, oligodendrocyte progenitor cells, oligodendrocytes, endothelial cells, ‘mural’ cells (pericytes or smooth muscle) and microglia. The genes used for identification are included in Supplementary Table 2 and a clustering of a subset of these genes is presented in Extended Data Fig. 1c.

Consistent with our previous study^48^, we did not observe sufficient differences in transcriptomic signatures to separate astrocytes from qNSCs and aNSCs from NPCs. We have described these clusters as ‘astrocyte-qNSCs’ and ‘aNSC-NPCs’ throughout this study. Some cell types were not identified when using the LMO protocol (for example, T cells), probably because cells such as T cells are small and their membranes may not allow for efficient LMO labeling. We also identified only a few ependymal cells in several of our datasets, although these cells are known to be numerous in the SVZ neurogenic niche. This is probably because ependymal cells are too big to be efficiently uploaded in droplets and/or they are sheared in the 10x microfluidic device.

### Cell cycle annotation and proliferative fraction

For cell cycle annotation (G1, S, G2/M) of cells in the SVZ neurogenic niche, we used Seurat’s CellCycleScoring function with default parameters. This annotation was used to calculate the ‘proliferative fraction’ in the SVZ neurogenic niche, that is, the percentage of cells predicted to be in S or G2/M phase. We used the proliferative fraction (ProliferativeFraction) as a functional metric of the SVZ neurogenic niche and used it to define ‘biological age’ in this study (‘Age prediction and validation strategy’).

To test the correlation between chronological age and proliferative fraction in the SVZ neurogenic niche, we used Pearson’s correlation. There was a negative correlation (Pearson *R* = −0.8) between chronological age and proliferative fraction in the SVZ.

### Age prediction and validation strategy

Chronological or biological age (‘label’) was regressed onto all log-normalized gene expression values ln((gene transcripts / cell transcripts) × 10,000) (‘features’) in a particular cell type using the R package glmnet (version 4.0.2)^51^. To determine the most robust method to predict age from single-cell RNA-seq data, we tested various preprocessing approaches: SingleCell, Pseudobulk, BootstrapCell (‘BoostrapCell preprocessing’) and EnsembleCell (‘EnsembleCell preprocessing’). SingleCell uses bona fide single-cell transcriptomes with minimal processing as input to a lasso regression model to predict chronological or biological age. Pseudobulk involves naïve pseudobulking all cells from the same cell type and sample before using a lasso regression model to predict chronological or biological age. BoostrapCell uses lasso regression models and EnsembleCell uses elastic net models (described separately below)^53^. There was no manual filtering of genes. Both lasso regression and elastic net regression enforce sparsity in the model coefficients with tunable parameter such that only a subset of genes will have nonzero coefficients in the trained aging clock models.

Chronological age was defined as months since birth. Biological age was defined as 35 – (ProliferativeFraction × 100) where ProliferativeFraction was the number of cells predicted to be in S or G2/M phase divided by the total number of cells from that sample. The number 35 was selected to transform biological age into the same range as chronological age.

For validation, models were built on 3 of the 4 cohorts of mice, and validation was done on the remaining cohort (stringent ‘leave-one-cohort-out’ validation (cross-cohort validation)). For training of each model, hyperparameters were optimized with fivefold to tenfold cross validation. To quantify the performance of the models, the data were presented as a correlation between the actual chronological (or biological) age of the mouse from which the cell originated (*x* axis) and the median predicted chronological (or biological) age for that mouse (*y* axis). Density of cells is represented with graded colors and each mouse is represented as a dot. We fitted a linear model (black line) through the points as well as the 95% confidence interval (light gray) using geom_smooth (ggplot2). Pearson’s correlation (*R*) is indicated on the graph. In dot plots, both the *R* values and the MAE, that is, median absolute error across all the cells, are presented.

To test the correlation between chronological age and biological age, we used the Pearson correlation. There was a positive correlation (*R* = 0.84) between chronological age and biological age predictions.

### BootstrapCell preprocessing

BootstrapCell uses a lasso model with the following characteristics: To generate a BootstrapCell, 15 single-cell transcriptomes were sampled without replacement from the pool of cells of a given cell type from a given animal (for example, oligodendrocytes from a single mouse). Gene counts were then summed. A BootstrapCell constructed from 15 cells was empirically found to balance the tradeoff between sample number and gene coverage per sample. This bootstrapping process was repeated 100 times for each cell type–animal combination. BootstrapCells were used as input into lasso regression models. This approach had the effect of normalizing the contribution of each animal rather than each single-cell transcriptome.

### EnsembleCell preprocessing

We devised and evaluated a second preprocessing and age prediction technique to compare to our BootstrapCell approach and to test robustness to changes in preprocessing and model architecture. In the EnsembleCell approach, 20 elastic net models were trained for each cell type. For each model, gene expression data from cells were randomly partitioned into groups of 15 single-cell transcriptomes and the unique transcript counts for all cells in each group were summed to create ‘EnsembleCells’. To predict age from the gene expression profile of a cell, we used the weighted average of predictions across all 20 models, where weights were determined by the *R*^2^ (coefficient of determination) of the model on a held-out validation set (‘Age prediction and validation strategy’).

### Use of aging clocks on independent mouse datasets

We determined if the single-cell-based models (‘aging clocks’) generated from our mouse SVZ neurogenic niche dataset could be applied to cells from an independent dataset and even to cells from another neurogenic region in the brain. To this end, we used a single-cell RNA-seq dataset of the SVZ neurogenic niche from young and old mice^48^ and a single-cell RNA-seq dataset of the dentate gyrus of the hippocampus from mice of three different ages^60^. These datasets were preprocessed as described above using the ‘BootstrapCell’ method. We examined the distribution of the predicted chronological or biological ages of each cell in these datasets, color coded by the age of the mouse of origin.

### Use of aging clocks on human datasets

To determine if the single-cell-based aging clocks generated from the mouse SVZ neurogenic niche could apply to cells from other regions of the brain and in other species, we used a single-nucleus RNA-seq dataset of the middle temporal gyrus from humans of different ages^61^. The dataset was preprocessed using the ‘BootstrapCell’ method as described above. As oligodendrocytes and astrocytes were present both in the human dataset and our mouse SVZ neurogenic niche dataset, we applied our oligodendrocyte and astrocyte-qNSC chronological aging clocks to the corresponding cell types in the human dataset. We rescaled the raw predictions linearly to obtain rescaled predicted chronological ages for each human BootstrapCell (rescaled predicted age = *m* × raw predicted age + b, where *m* = 10 and *b* = 125.5 for oligodendrocytes; *m* = 5 and *b* = 32.75 for astrocytes). The linear rescaling did not change the reported correlation between predicted chronological age and actual chronological age. Correlation plots were generated as described in ‘Age prediction and validation strategy’.

### Cell-type-specific aging clocks using Tabula Muris Senis

To determine whether the method we used to derive cell-type-specific aging clocks was generalizable to tissues other than neurogenic niches, we used the count matrices from the single-cell RNA-seq dataset of the multi-tissue aging atlas Tabula Muris Senis^62^. We chose three diverse cell types in different tissues: endothelial cells from limb muscle, mature natural killer T cells from spleen and podocytes from kidney. For each cell type, the data were preprocessed and aging clocks were trained using the BootstrapCell approach described above. The performance of these models was evaluated by iteratively training on all mice except for one mouse and obtaining predictions on the held-out mouse (‘leave-one-mouse-out’ cross validation (cross-mouse validation) instead of ‘leave-one-cohort-out’ cross validation (cross-cohort validation) because there were no distinct cohorts in this dataset).

### Identification of genes that contribute to the aging clocks

Genes that contribute to each aging clock model were retrieved by selecting all genes from the clocks with nonzero coefficients (Supplementary Table 4). The weight of a gene on each clock model (that is, the level of contribution based on coefficient values) and the sign of the coefficient (positive, higher gene expression is associated with older age; negative, lower gene expression is associated with older age) are indicated using a donut plot, with sector size indicating the gene weight and color indicating coefficient sign. Genes with positive coefficient are mostly upregulated with age, and genes with negative coefficient are mostly downregulated with age. The regulation of each chronological and biological clock gene (compared to other genes) is presented using a volcano plot (Extended Data Fig. 4). Most genes selected by the clocks were differentially expressed during aging. Less than half of the genes selected by chronological and biological aging clocks in a particular cell type overlapped (Supplementary Table 4). To determine if chronological or biological clock genes were shared across cell types or specific to each cell types, we used UpSet plots. Most genes selected by chronological or biological clocks were cell-type specific. The ‘impact’ (sum of absolute values of coefficient) and ‘count’ (sum of gene number) of shared genes or specific genes are indicated as a stacked bar plot.

### Properties of genes that contribute to the aging clocks

To determine if genes that contribute to the aging clocks have specific properties, we examined their variability by plotting the coefficient of variation as a function of mean expression. Genes used by the clocks were more highly expressed and, at a given level of expression, had a higher coefficient of variation (that is, were more variable) than genes not in the clock (Extended Data Fig. 3a).

We also verified that the increased variability of genes that contribute to the clocks was not merely due to sparsity in the single-cell RNA-seq dataset. On average, the majority of cells (for each cell type) express the genes that contribute to the clocks and this is higher than what was observed for genes that do not contribute to the clock (Extended Data Fig. 3b).

### Gene-set enrichment analysis

GSEA was performed using Enrichr^108^ to query cell-type-specific clock genes for enrichment against GO biological process gene sets. Statistics were exported from the Enrichr web tool and processed and visualized in R with ggplot2 (version 3.3.3) package.

### Parabiosis cohorts and single-cell RNA-seq dataset

Two independent cohorts of heterochronic parabiosis were generated (cohort 1 and cohort 2). Parabiosis cohort 1 involved six male mice across three pairings. We collected SVZ niches from one isochronic young mouse (5 months, control), one heterochronic young mouse (5 months, old blood), one heterochronic old mouse (26 months, young blood) and one isochronic old mouse (26 months, control), for a total of four SVZ niches (of six mice). Old parabionts were C57BL/6 male mice from the NIA Aged Rodent colony at Charles River. Young parabionts were B6.SJL-*Ptprc*^*a*^ *Pepc*^*b*^/BoyJ male (Pep boy) mice from The Jackson Laboratory and C57BL/6 male mice from the NIA. Of the young, only the Pep boy mice were used for transcriptomics. Congenic (rather than isogenic) pairings were performed to enable verification of blood chimerism by FACS with antibodies specific to CD45.1 (BioLegend, 110705; 1:100 dilution) or CD45.2 (BioLegend, 109814; 1:100 dilution) alleles. Mice were 4 and 25 months old at the start of the experiment, and parabiosis was conducted for 5 weeks until cell collection, when mice were 5 and 26 months old. Pairs were established as previously described^69,75,80^ by suturing the peritoneums of adjacent flanks and joining skin with surgical clips. Five weeks after the parabiosis surgery, mice were anaesthetized with 2.5% vol/vol avertin, euthanized by cardiac puncture and perfused with 15 ml PBS with heparin (50 U ml^−1^). SVZ dissection, digestion and FACS were performed as describe above. 10x Genomics single-cell transcriptome V2 libraries (one sample per 10x lane) were generated and sequenced on one Illumina HiSeq lane by the Stanford Function Genomics Facility. Animal care and parabiosis procedures were performed in accordance with Stanford University under IACUC protocols 8661 and 16246.

Parabiosis cohort 2 involved eighteen male mice across nine pairings. We collected SVZ niches from four isochronic young mice (5 months, control), four heterochronic young mice (5 months, old blood), four heterochronic old mice (21 months, young blood) and six isochronic old mice (21 months, control), for a total of eighteen SVZ niches (of eighteen mice). All mice in this cohort were sourced from the Jackson Laboratory and housed in the Veterinary Medical Unit at the Palo Alto VA^77^. Old mice were C57BL/6J and young were C57BL/6J or C57BL/6-Tg(UBC-GFP)30Scha/J. Mice were aged 4 and 19.5 months at the start of the experiment, and parabiosis proceeded for 5 weeks until cell collection, when mice were 5 and 21 months old. Surgeries were performed as described above. Five weeks after surgery, mice were anesthetized with 2.5% vol/vol avertin, euthanized by cardiac puncture and perfused with 15 ml PBS with heparin (50 U ml^−1^). SVZ dissection, digestion and FACS were performed as describe above. Tissue collection took place on three separate days and samples were multiplexed with LMOs. 10x Genomics single-cell transcriptome V3 libraries were generated in-house and sequenced by Novogene on an Illumina NovoSeq lane. Animal care and parabiosis procedures were approved by the VA Palo Alto Committee on Animal Research and listed on ACORP LUO1736.

Parabiosis cohort 1 and cohort 2 were generated in different animal facilities, by different surgeons, in different years, and they were analyzed with different versions of 10x Genomics single-cell transcriptomics kits. For visualization, data from the two independent cohorts were integrated on the cohort identity using the RunHarmony command from Harmony^109^. There were no statistically significant differences between young isochronic (control) predicted chronological ages across cohorts in all six cell-type-specific aging clocks (Wilcoxon rank-sum test for median predicted chronological ages), suggesting that there was not a major batch effect that could have influenced the age prediction.

### Exercise cohort and single-cell RNA-seq dataset

C57BL/6 male mice from the NIA Aged Rodent colony at Charles River were housed in the Veterinary Medical Unit at the Palo Alto VA^97^. Young and old mice were aged 4.5 months and 21.5 months, respectively, at the start of the 5-week voluntary wheel running intervention, so they were 6 months and 23 months when tissues were collected. During the intervention period, mice (*n* = 4 for each age group) were singly housed in cages accommodating a running wheel. Control mice (*n* = 3–4 for each age group) had no access to a wheel. Running was verified by recording wheel revolutions. After 5 weeks, mice were anaesthetized with 2.5% v/v avertin, euthanized by cardiac puncture, perfused and cell suspensions from dissected SVZs generated as described in ‘Tissue and cell collection for the SVZ neurogenic niche’. Next, 10x Genomics V3.0 transcriptomics kits were used to generated libraries without upstream sample multiplexing. Tissue processing occurred across two separate mornings. SVZ libraries were pooled and sequenced on an Illumina NovoSeq.

### Effect of rejuvenation interventions on the aging clocks

To measure the effect of heterochronic parabiosis and exercise on the aging clocks, we examined the distribution of predicted chronological or biological ages as described in ‘Use of aging clocks on independent mouse datasets’. We calculated the effect by the difference in median predicted chronological or biological age between intervention and control. In dot plots, these differences were represented as ‘effect’, using size and intensity of color, with blue indicating ‘rejuvenation’ and red indicating ‘aging’.

### Comparison of heterochronic parabiosis and exercise effects

To compare the effect of heterochronic parabiosis and exercise, we calculated the mean of the difference between the median predicted chronological age for a mouse for each intervention (data from cohort 1 and cohort 2 for heterochronic parabiosis). Genes that were reversed by each intervention or by both, based on direction of average log fold change, were identified.

### Differential expression analysis

To determine genes that were impacted by different interventions independently of the aging clocks, we used differential expression analysis, focusing on aNSC-NPCs (as this cell type is impacted by both interventions). MAST^110^ software was used to calculate differential expression statistics between three different conditions: age (young versus old), young blood (heterochronic parabiosis versus isochronic old control), exercise (exercise versus sedentary in old mice). To determine the DEGs between young and old, we defined ‘young’ as mice <7 months and ‘old’ as mice >20 months. Permissive cutoffs of 1.1-fold change and FDR < 0.1 were applied in each of the three different conditions. Overlap was presented as a Venn diagram.

### Gene signature analysis

For specific gene signature analysis, we summed the expression of genes in one cell type from single-cell transcriptomic datasets within a specific gene signature defined by a specific GO term. Among the different signatures tested, we selected those that were significantly increased with age and reversed by at least one intervention. We focused on two signatures: the ‘interferon-γ response’ signature defined as the sum of all normalized expression values of genes in the interferon gene set defined by Dulken et al.^48^ and the ‘negative regulation of neurogenesis’ gene signature defined as the sum of all normalized expression values of genes in the GO term ‘negative regulation of neurogenesis’ gene set (v6.21)^111^. Data were presented as violin plots and statistical analyses were performed using the Wilcoxon rank-sum test at the cell level.

### Intervention classification models

To evaluate the aging relevance of ‘rejuvenation’ interventions, we generated cell-type-specific models trained on the intervention rather than age as a label. We used classification models, based on logistic regression (cv.glmnet(type.measure = ‘mse’, family = ‘binomial’) using all log-normalized gene expression values ln((gene transcripts / cell transcripts) × 10,000) as features. These intervention classification models were trained on single-cell RNA-seq data from heterochronic parabiosis (young blood) versus isochronic parabiosis old (control) or from exercise versus sedentary old mice. The data were preprocessed using the same BootstrapCell approach as described above. For logistic regression, the label used corresponded to either the intervention (‘0’) or control (‘1’). Cross validation was performed on held-out cells (25% of the cells that were not used to build the models). After training and validating the intervention classification models, we applied these models to the single-cell RNA-seq dataset of the SVZ neurogenic niche from 28 mice, tiling 26 ages from young (3.3 months) to old (29 months). Data were plotted as described in ‘Age prediction and validation strategy’, with (log(*p*(control) / *p*(intervention))) as a function of the actual chronological age of aNSC-NPC BootstrapCell transcriptomes. Old mice were more likely to be classified as ‘isochronic old control’, whereas young mice were more likely to be classified as ‘heterochronic old’, indicating that the gene signature that distinguishes exposure to young and old blood is relevant to aging. *R* is the Pearson correlation. Higher correlation indicates that the main intervention signature overlaps with and reverses age-related changes. Correlations between intervention state prediction and chronological age across cell types and interventions were assessed, with a separate classifier built for each. The exercise classifiers were built to distinguish old sedentary from old exercised transcriptomes for each cell type. The lower correlation between intervention state predictions and age for the exercise samples implies that the signatures that distinguishes exercised and sedentary mice are less related to aging than those derived from parabiosis intervention classifiers.

### Statistics and reproducibility

No statistical methods were used to predetermine sample sizes; we determined our sample sizes based on our previous analysis of similar types of datasets^48^. For study design, we used four independent cohorts of mice, each spanning different ages, to build the age prediction models. This design allows us to test the machine learning aging clock models with a robust cross-cohort validation (that is, ‘leave-one-cohort-out’ validation). Two independent experiments of heterochronic parabiosis were performed, involving 6 mice (4 collected, cohort 1) and 18 mice (cohort 2), with data collection spread across 4 d. One experiment of exercise (with controls lacking a running wheel) was performed, involving 15 mice processed across 2 d. Animals from group 3 from parabiosis cohort 2 were excluded because sample multiplexing failed and it was not possible to distinguish samples. The experiments were not randomized. Investigators were not blinded to allocation during experiments and outcome assessment, although the genomics analyses were performed in a systematic manner. To test correlations, we used Pearson’s correlation. To determine the statistical significance of the differences between intervention and control, we used the Wilcoxon rank-sum test (a non-parametric test).

### Reporting summary

Further information on research design is available in the Nature Portfolio Reporting Summary linked to this article.

## Supplementary information

Reporting Summary

Supplementary Table 1 Mice metadata including age and multiplexing oligonucleotide sequence.

Supplementary Table 2 Gene markers for cell-type identification along with statistical significance and effect size measures from Seurat.

Supplementary Table 3 Prediction performance summary table for all models.

Supplementary Table 4 Clock genes and coefficients for all models.

Supplementary Software 1 Zip file containing a frozen version of the code used in this paper.

## Data Availability

All raw sequencing reads and key processed files are accessible at BioProject PRJNA795276 (aging, parabiosis) and the Gene Expression Omnibus under accession GSE196364 (exercise). Processed data files for the aging and parabiosis data can be found at 10.5281/zenodo.7145399. Processed data files for the exercise data can be found at 10.5281/zenodo.7338746. External raw sequencing reads for the mouse hippocampus dataset are accessible at the Gene Expression Omnibus under accession GSE159768. External data on human middle temporal gyrus are accessible at https://portal.brain-map.org/atlases-and-data/rnaseq/human-mtg-smart-seq/. External data from Tabula Muris Senis are accessible at https://figshare.com/projects/Tabula_Muris_Senis/64982/. PanglaoDB can be accessed at https://panglaodb.se/.

## Code availability

The code used to analyze genomic data and generate aging clocks in the current study is available in the GitHub repository for this paper (https://github.com/sunericd/svz_singlecell_aging_clocks/). A frozen version of the code repository is available as Supplementary Software 1.
