# Supplementary material for: Cell-type-specific aging clocks to quantify aging and rejuvenation in neurogenic regions of the brain
Source: Nat Aging. 2022 Dec 19;3(1):121–37. doi: 10.1038/s43587-022-00335-4 (PMC10154228; doi:10.1038/s43587-022-00335-4)
Supplement: Supplementary file 1 — Reporting Summary [file 43587_2022_335_MOESM1_ESM.pdf]

nature portfolio

Corresponding author(s): Anne Brunet

Last updated by author(s): Feb 3, 2022

Reporting Summary

Nature Portfolio wishes to improve the reproducibility of the work that we publish. This form provides structure for consistency and transparency in reporting. For further information on Nature Portfolio policies, see our Editorial Policies and the Editorial Policy Checklist.

Statistics

For all statistical analyses, confirm that the following items are present in the figure legend, table legend, main text, or Methods section.

n/a	Confirmed	
☐	☒	The exact sample size (*n*) for each experimental group/condition, given as a discrete number and unit of measurement
☐	☒	A statement on whether measurements were taken from distinct samples or whether the same sample was measured repeatedly
☐	☒	The statistical test(s) used AND whether they are one- or two-sided<br>*Only common tests should be described solely by name; describe more complex techniques in the Methods section.*
☐	☒	A description of all covariates tested
☐	☒	A description of any assumptions or corrections, such as tests of normality and adjustment for multiple comparisons
☐	☒	A full description of the statistical parameters including central tendency (e.g. means) or other basic estimates (e.g. regression coefficient) AND variation (e.g. standard deviation) or associated estimates of uncertainty (e.g. confidence intervals)
☐	☒	For null hypothesis testing, the test statistic (e.g. *F*, *t*, *r*) with confidence intervals, effect sizes, degrees of freedom and *P* value noted<br>*Give P values as exact values whenever suitable.*
☒	☐	For Bayesian analysis, information on the choice of priors and Markov chain Monte Carlo settings
☒	☐	For hierarchical and complex designs, identification of the appropriate level for tests and full reporting of outcomes
☐	☒	Estimates of effect sizes (e.g. Cohen's *d*, Pearson's *r*), indicating how they were calculated

Our web collection on statistics for biologists contains articles on many of the points above.

Software and code

Policy information about availability of computer code

Data collection	No software was used.
Data analysis	CellRanger (3.0.2) and custom analyses in R (3.6.3) using several packages: Seurat (3.2.3), glmnet (4.0), tidyverse (1.3.0), MAST (1.10.0), sctransform (0.3.2), harmony (1.0), ggpubr (0.4.0), ggplot2 (3.3.5). Web version of Enrichr (accessed after March 29, 2021 update) through https://maayanlab.cloud/Enrichr/. Analysis code can will be available at https://github.com/sunericd/svz_singlecell_aging_clocks. For reviewers the code can be downloaded from https://www.dropbox.com/s/hxd4xzn11wxe52h/svz_singlecell_aging_clocks-main.zip?dl=0.

For manuscripts utilizing custom algorithms or software that are central to the research but not yet described in published literature, software must be made available to editors and reviewers. We strongly encourage code deposition in a community repository (e.g. GitHub). See the Nature Portfolio guidelines for submitting code & software for further information.

Data

Policy information about availability of data

All manuscripts must include a data availability statement. This statement should provide the following information, where applicable:

- Accession codes, unique identifiers, or web links for publicly available datasets
- A description of any restrictions on data availability
- For clinical datasets or third party data, please ensure that the statement adheres to our policy

All raw sequencing reads and key processed files are accessible at BioProject PRJNA795276 (Aging, Parabiosis) and Gene Expression Omnibus GSE196364 (Exercise, a secure token can be obtained from Dr. Ling Liu (ref 97): lingliu@stanford.edu)). Processed data files for the Aging and Parabiosis data can be found at https://doi.org/10.5281/zenodo.7145399. External raw sequencing reads for the mouse hippocampus dataset are accessible at Gene Expression Omnibus GSE159768.

External data on human middle temporal gyrus is accessible at https://portal.brain-map.org/atlases-and-data/rnaseq/human-mtg-smart-seq. External data from Tabula Muris Senis is accessible at https://figshare.com/projects/Tabula_Muris_Senis/64982. PanglaoDB can be accessed at https://panglaodb.se/.

Field-specific reporting

Please select the one below that is the best fit for your research. If you are not sure, read the appropriate sections before making your selection.

☒ Life sciences ☐ Behavioural & social sciences ☐ Ecological, evolutionary & environmental sciences

For a reference copy of the document with all sections, see nature.com/documents/nr-reporting-summary-flat.pdf

Life sciences study design

All studies must disclose on these points even when the disclosure is negative.

Sample size	No statistical methods were used to pre-determine sample sizes; we determined our sample sizes based on our previous analysis of similar types of datasets (Dulken et al, 2019).
Data exclusions	Animals from group 3 from parabiosis cohort 2 was excluded because sample multiplexing failed and it was impossible to distinguish samples.
Replication	For study design, we used four independent cohorts of mice, each spanning different ages, to build the age prediction models. This design allows us to test the machine learning aging clock models with a robust cross-cohort validation (i.e. “leave one-cohort-out” validation). Two independent experiments of heterochronic parabiosis were performed, involving 6 mice (4 collected, cohort 1) and 18 mice (cohort 2) data collection across spread across 4 days. One experiment of exercise (with controls lacking a running wheel) was performed, involving 15 mice processed across 2 days.
Randomization	Experiments were not randomized.
Blinding	Investigators were not blinded to allocation during experiments and outcome assessment, though the genomics analyses were done in a systematic manner.

Reporting for specific materials, systems and methods

We require information from authors about some types of materials, experimental systems and methods used in many studies. Here, indicate whether each material, system or method listed is relevant to your study. If you are not sure if a list item applies to your research, read the appropriate section before selecting a response.

Materials & experimental systems

n/a	Involved in the study	
☐	☒	Antibodies
☒	☐	Eukaryotic cell lines
☒	☐	Palaeontology and archaeology
☐	☒	Animals and other organisms
☒	☐	Human research participants
☒	☐	Clinical data
☒	☐	Dual use research of concern

Methods

n/a	Involved in the study	
☒	☐	ChIP-seq
☐	☒	Flow cytometry
☒	☐	MRI-based neuroimaging

Antibodies

Antibodies used	CD45.1 (BioLegend 110705, 1:100) or CD45.2 (BioLegend 109814, 1:100) (to verify parabiosis chimerism for parabiosis cohort 1)
Validation	All antibodies were validated for the indicated applications by the manufacturer.

Animals and other organisms

Policy information about studies involving animals; ARRIVE guidelines recommended for reporting animal research

Laboratory animals	For aging cohorts and exercise cohort, mice used were male C57BL/6 mice obtained from the NIA Aged Rodent colony. For parabiosis cohort 1, old mice were male C57BL/6 mice from the NIA Aged Rodent colony and young mice were male B6.SJL-Ptprca Pepcb/BoyJ male (Pep boy) from the Jackson Lab. For parabiosis cohort 2, old mice were male C57BL/6J and young mice were male C57BL/6J or C57BL/6-Tg(UBC-GFP)30Scha/J from The Jackson Laboratory. A table with further mouse details is available in Supplementary Table 1. Mice were housed at ~21 celsius degree with 50% humidity.
Wild animals	No wild animals were used in the study.

Field-collected samples | No field-collected samples were used in the study.

Ethics oversight | All experiments were done with approval at Stanford University under IACUC protocols 8661 and 16246.

Note that full information on the approval of the study protocol must also be provided in the manuscript.

Flow Cytometry

Plots

Confirm that:

☐ The axis labels state the marker and fluorochrome used (e.g. CD4-FITC).

☐ The axis scales are clearly visible. Include numbers along axes only for bottom left plot of group (a 'group' is an analysis of identical markers).

☐ All plots are contour plots with outliers or pseudocolor plots.

☐ A numerical value for number of cells or percentage (with statistics) is provided.

Methodology

Sample preparation | The SVZ from each hemisphere was micro-dissected and dissociated with enzymatic digestion with papain at a concentration of 14 U ml−1, rocking for 10 min at 37°C. The dissociated SVZ was then triturated in a solution containing 0.7 mg ml−1 ovomucoid and 0.5 mg ml−1 DNaseI (Sigma-Aldrich, DN25-100MG) in DMEM/F12 (Thermo Fisher, 11330032). The dissociated cells from the SVZ were then centrifuged through 22% Percoll (Sigma-Aldrich, GE17-0891-01) in PBS to remove myelin debris. After centrifugation, cells were filtered through a 35-μm snap-cap filter (Corning, 352235), washed once with 1.5 ml of FACS buffer (HBSS (Thermo-Fisher, 14175103), 1% bovine serum albumin (Sigma, A7979), 0.1% glucose (Sigma-Aldrich, G7021-1KG) and spun down for 5 min at 300g. Cells were then resuspended in 120 μl FACS buffer with live/dead staining was performed using 1 μg ml−1 propidium iodide (BioLegend, 421301) and kept on ice until sorting. FACS sorting was performed on a BD FACS Aria II sorter, using a 100-μm nozzle at 13.1 PSI. Cells were sorted into low protein binding microcentrifuge tubes containing 750 μl of PBS with 1% BSA and 0.1% glucose.

Instrument | BD FACS Aria II housed in the Stanford Shared FACS Facility.

Software | No FACS data analysis was reported. Sorting was only used to enrich live cells in single cell RNA-seq dataset and to verify chimerism of parabiosis mice cohort 1.

Cell population abundance | Sorting was only used to enrich live cells. Between 15,000 and 80,000 live cells were typically isolated from each SVZ.

Gating strategy | Debris, doublets, and PI-positive (dead) cells were removed.

☐ Tick this box to confirm that a figure exemplifying the gating strategy is provided in the Supplementary Information.
